# Coupling of a Core Post-Translational Pacemaker to a Slave Transcription/Translation Feedback Loop in a Circadian System

**DOI:** 10.1371/journal.pbio.1000394

**Published:** 2010-06-15

**Authors:** Ximing Qin, Mark Byrne, Yao Xu, Tetsuya Mori, Carl Hirschie Johnson

**Affiliations:** 1 Department of Biological Sciences, Vanderbilt University, Nashville, Tennessee, United States of America; 2 Physics Department, Spring Hill College, Mobile, Alabama, United States of America; Rijksuniversiteit Groningen, The Netherlands

## Abstract

Cyanobacteria are the only model circadian clock system in which a circadian oscillator can be reconstituted in vitro. The underlying circadian mechanism appears to comprise two subcomponents: a post-translational oscillator (PTO) and a transcriptional/translational feedback loop (TTFL). The PTO and TTFL have been hypothesized to operate as dual oscillator systems in cyanobacteria. However, we find that they have a definite hierarchical interdependency—the PTO is the core pacemaker while the TTFL is a slave oscillator that quickly damps when the PTO stops. By analysis of overexpression experiments and mutant clock proteins, we find that the circadian system is dependent upon the PTO and that suppression of the PTO leads to damped TTFL-based oscillations whose temperature compensation is not stable under different metabolic conditions. Mathematical modeling indicates that the experimental data are compatible with a core PTO driving the TTFL; the combined PTO/TTFL system is resilient to noise. Moreover, the modeling indicates a mechanism by which the TTFL can feed into the PTO such that new synthesis of clock proteins can phase-shift or entrain the core PTO pacemaker. This prediction was experimentally tested and confirmed by entraining the in vivo circadian system with cycles of new clock protein synthesis that modulate the phosphorylation status of the clock proteins in the PTO. In cyanobacteria, the PTO is the self-sustained core pacemaker that can operate independently of the TTFL, but the TTFL damps when the phosphorylation status of the PTO is clamped. However, the TTFL can provide entraining input into the PTO. This study is the first to our knowledge to experimentally and theoretically investigate the dynamics of a circadian clock in which a PTO is coupled to a TTFL. These results have important implications for eukaryotic clock systems in that they can explain how a TTFL could appear to be a core circadian clockwork when in fact the true pacemaker is an embedded biochemical oscillator.

## Abstract 2

Analysis of the cyanobacterial circadian biological clock reveals a complex interdependence between a transcription/translation feedback loop and a biochemical oscillator.

## Author Summary

Many organisms from bacteria to humans have evolved circadian mechanisms for regulating biological processes on a daily time scale. In cyanobacteria, a minimal system for such cyclical regulation can be reconstituted in vitro from three proteins, called KaiA, KaiB, and KaiC. This three-protein oscillator is believed to regulate the cyclical activities in vivo through a post-translational mechanism that involves rhythmic phosphorylation of KaiC. Although this post-translational oscillator (PTO) is sufficient for generating rhythms in vitro, the cyanobacterial circadian system in vivo also includes a transcriptional/translational feedback loop (TTFL). The precise roles of the PTO and the TTFL and their interdependence in forming the complete clock system in vivo are unclear. By manipulating wild-type and mutant clock protein expression in vivo, we here show that the cyanobacterial circadian system is dependent upon the biochemical oscillator provided by the PTO and that suppression of the PTO leads to a residual damped (slave) oscillation that results from the TTFL. Mathematical modeling shows that the experimental data are compatible with a mechanism in which the PTO acts as a pacemaker to drive the activity of the TTFL. Moreover, our analyses suggest a mechanism by which the TTFL can feed back into the PTO such that new synthesis of the Kai proteins entrains the core PTO pacemaker. Therefore, the PTO and TTFL appear to have a definite hierarchical interdependency: the PTO is a self-sustained core pacemaker that can oscillate independently of the TTFL, but the TTFL is a slave oscillator that damps when the phosphorylation status of KaiC in the PTO is clamped. The core circadian pacemaker in eukaryotes is thought to be a TTFL, but our results with cyanobacteria have important implications for eukaryotic clock systems in that they can explain how a TTFL could appear to be the core clock when in fact the true pacemaker is an embedded biochemical oscillator.

## Introduction

The mechanism of circadian (daily) clocks in eukaryotes is generally thought to be based upon autoregulatory transcriptional/translational feedback loops (TTFLs) [1],[2]. When the essential components of the circadian clock in the prokaryotic cyanobacterium *Synechococcus elongatus* were identified [3] as the proteins KaiA, KaiB, and KaiC, the initial interpretation that the core of this prokaryotic clockwork might also be a TTFL was based on the same kind of evidence that supports TTFL oscillators in eukaryotes, namely: (i) rhythms of abundance for mRNAs and proteins encoded by “clock genes,” (ii) feedback of clock proteins on their gene's transcription, and (iii) phase setting by experimental expression of clock proteins [3]–[5]. However, later studies found data that were inconsistent with a core TTFL oscillator in cyanobacteria; e.g., global inhibition of transcription and translation had little effect upon the circadian rhythm of KaiC phosphorylation [6], and the promoters driving *kaiBC* gene expression could be replaced with non-specific heterologous promoters without disturbing the circadian rhythm [7],[8]. Moreover, prolonged treatments of cyanobacterial cells with the protein synthesis inhibitor chloramphenicol did not perturb the phase of the circadian system after return to normal conditions [4],[9]. Then in 2005 came the astonishing discovery that the phosphorylation status of KaiC in vitro continued to cycle when the three Kai proteins were combined in a test tube with ATP [10]. This in vitro rhythm persists with a circa-24 h period for at least 10 d and is temperature compensated [10],[11], which indicates that a circadian temperature compensation mechanism is also encoded in the characteristics of the three Kai proteins and the nature of their interactions. In vivo, this three-protein biochemical oscillator operates as a post-translational oscillator (PTO) [6],[10]. Clearly, a TTFL is not necessary for the circadian rhythm of KaiC phosphorylation.

The PTO manifests itself in vitro as three different rhythms that are probably interrelated. The first is the originally observed rhythm of KaiC phosphorylation [10]. The second is a rhythm of formation of complexes among KaiA, KaiB, and KaiC [12],[13], and the third is a rhythm of ATP hydrolysis [14]. At this time, it is not clear which of these rhythms is the most fundamental to the PTO mechanism or whether they are all so tightly intermeshed as to be inseparable. Some or all of these rhythmic processes may also be involved in the control of outputs through interactions with other proteins such as SasA and/or RpaA [15]. Moreover, while each of these three rhythms can be measured in vitro, only the KaiC phosphorylation rhythm can be monitored in vivo (as a rhythmic shift of KaiC mobility on immunoblots). Therefore, in this paper, the phosphorylation rhythm will be taken as the indicator of the PTO in vivo.

Since the *kaiABC* gene cluster is essential for rhythms in vivo and the rhythm of KaiC phosphorylation could run without a TTFL in vitro [10] and in vivo [4],[6], those results implied that the KaiABC oscillator was the self-sustained core pacemaker and that transcription and translation was involved only in the output [6],[10]. More recently, however, Kitayama and coworkers suggested that “transcription- and translation-based oscillations in KaiC abundance are also important for circadian rhythm generation in cyanobacteria” [16]. First, those authors reported that over-expression of KaiA resulting in constitutively hyper-phosphorylated KaiC (a “clamp” of KaiC phosphorylation status) allows circadian rhythms of gene expression as monitored by promoter-driven luciferase reporters in vivo. Moreover, mutants of KaiC that mimicked constitutive hyperphosphorylation or hypophosphorylation allowed rhythms in vivo. The key phospho-regulatory sites on KaiC are S431 and T432 [17],[18]; Kitayama and coworkers reported that substitution of glutamate on those two residues (KaiC^EE^) created a constitutively hyper-phosphorylated KaiC mutant strain that “showed a dampened but clear rhythm with a period of 48 h” [16]. Because cyanobacterial cells apparently exhibited oscillations when the KaiABC oscillator was inactivated by clamping the phosphorylation status of KaiC, these two experimental approaches led Kitayama and coworkers to conclude that “transcription-translation oscillates even in the absence of the KaiC phosphorylation cycle and that this oscillation could persist regardless of the phosphorylation state and kinase activity of KaiC” [16]. These results therefore opened the possibility that the KaiABC oscillator (PTO) was not an obligatory core oscillator in cyanobacteria.

We have extended the experiments of Kitayama and coworkers, and our results lead us to different interpretations, namely that the TTFL is a damped slave oscillator while the PTO is the core pacemaker. Our results indicate that repression of the KaiC phosphorylation rhythm by KaiA overexpression strictly correlates with the suppression of the larger circadian system. We find that the rhythms generated by cells expressing KaiC^EE^ are clearly damped and of long period. Moreover, the damped rhythms exhibited by KaiC^EE^ are not compensated for metabolic activity and therefore cannot be considered as a bona fide circadian phenomenon. These results direct us towards a model of the entire system that explains how the core pacemaker can be a PTO while having input from a TTFL. The implications of this organization extend beyond the cyanobacterial case and encourage a re-evaluation of the evidence for a core TTFL in eukaryotic circadian clocks.

## Results

### The KaiC Phosphorylation Rhythm Is the Most Consistent Rhythm under Different In Vivo Conditions

**Figure 1:**
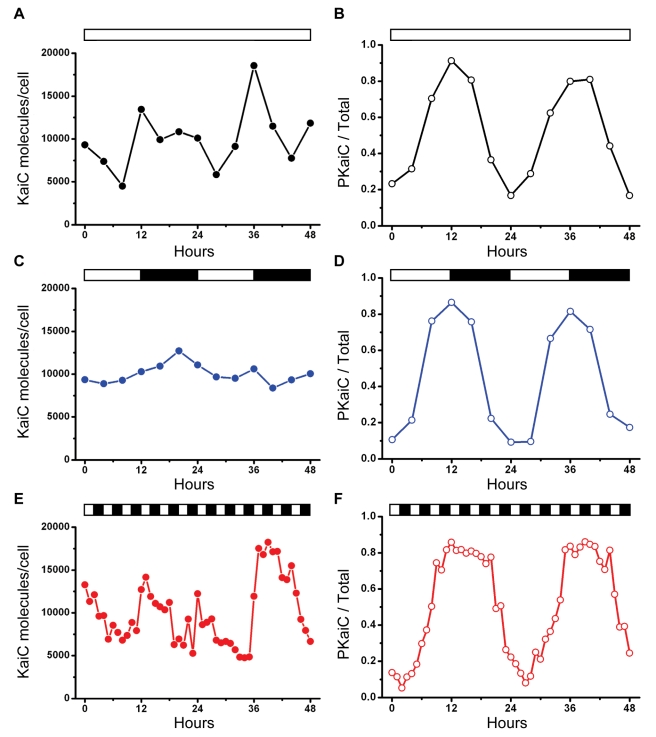
In vivo patterns of KaiC abundance and phosphorylation under different illumination conditions. WT cells (strain AMC149) were assayed in (A, B) LL (constant light; data are averages of three independent experiments), (C, D) LD12:12 (12 h light/12 h dark; data are averages of two independent experiments), (E, F) LD2:2 (2 h light/2 h dark). (A, C, E) KaiC abundance. Based on data of Kitayama and coworkers [30], the values are normalized to an average number of 10,000 KaiC molecules/cell. (B, D, F) KaiC phosphorylation status, expressed as a ratio of phosphorylated KaiC (P-KaiC) to total KaiC. The KaiC phosphorylation pattern is the most consistent rhythm among the three different in vivo conditions. White/black bars denote light/darkness. Raw data from representative experiments are shown in Figure S1A for KaiC abundance and Figure S1B for KaiC phosphorylation.

Compared with the rhythm of KaiC abundance that could be the result of a TTFL involving KaiC expression [4],[6], the KaiC phosphorylation rhythm is the most reproducible molecular rhythm that can be measured in vivo under a range of conditions in *S. elongatus*. In both LL and DD, KaiC phosphorylation is robustly rhythmic, despite the fact that KaiC abundance is rhythmic in LL but not in DD [6]. Figure 1 shows that KaiC abundance is rhythmic in LL as noted before [4] with a concomitant rhythm of KaiC phosphorylation (Figure 1A, 1B; see Figure S1A and S1B for representative immunoblots). However, in a light/dark cycle of 12 h light, 12 h dark ( = LD 12:12), there is not a clear daily rhythm of KaiC abundance, while the KaiC phosphorylation rhythm remains robust with a phase relationship that is similar to that in LL (Figures 1C, 1D, S1). The abundances of KaiA and KaiB can also be arhythmic in LD 12:12 (representative example shown in Figure S2). Note that LD 12:12 is more relevant to the organism in nature than LL, and yet there is no reproducible KaiC abundance rhythm that would be expected to result from a TTFL. The result of Figure 1C is initially inexplicable since global transcription is strongly dependent upon light in *S. elongatus* [6], and therefore a rhythm of KaiC abundance would be expected. However, we discovered that KaiC degradation is also strongly light-dependent; in darkness, KaiC degradation is inhibited (Figure S3). Therefore, synthesis and degradation of KaiC is counterbalanced in the light phase of LD, while KaiC is neither synthesized [6] nor degraded (Figure S3) in the dark phase of LD; consequently, KaiC abundance remains nearly constant in LD (Figure 1C). On the other hand, in LL the synthesis of KaiC is rhythmic but degradation continues in the subjective night, leading to a rhythm of KaiC abundance in LL [19].

To test whether the KaiC phosphorylation rhythm was disrupted by metabolic noise, we altered the environmental conditions to LD 2:2 (2 h light followed by 2 h darkness). Because *S. elongatus* is an obligate photoautotroph that is absolutely dependent upon photosynthesis for energy, a high frequency light/dark cycle will have major effects on intracellular photosynthesis, redox status, and metabolism. The 4 h cycle of LD 2:2 allowed the persistence of a circa-24 h rhythm of luminescence as a reporter of circadian gene expression (Figure S1C). Under these conditions, there is a noisy and possibly rhythmic pattern of KaiC abundance while there is a robust and clear rhythm of KaiC phosphorylation (Figure 1E and 1F). Therefore, it is the KaiC phosphorylation rhythm (an indicator of the PTO) that is the most reproducible rhythm under a wide range of in *vivo* conditions (LL, LD 12:12, and LD 2:2), not the KaiC abundance rhythm that could be a direct consequence of a TTFL oscillator.

### Constitutive Hyper-Phosphorylation of KaiC Disrupts the Normal Circadian System

As described in the Introduction, Kitayama and coworkers [16] concluded that “transcription-translation oscillates even in the absence of the KaiC phosphorylation cycle and that this oscillation can persist regardless of the phosphorylation state and kinase activity of KaiC” [16]. One of the main lines of evidence that they marshaled to support their conclusions was that constitutive hyper-phosphorylation of KaiC—either by over-expression of KaiA or by mutation of KaiC—allowed the in vivo system to operate relatively normally. In the in vitro system, a higher proportion of KaiA causes KaiC hyperphosphorylation and a suppression of the in vitro oscillation (Figure S4), so it is reasonable to hypothesize that KaiA overexpression in vivo will have the same effect. However, when we express KaiA in vivo, we find either different results and/or interpret the data from a different perspective than the report of Kitayama et al. [16].

**Figure 2:**
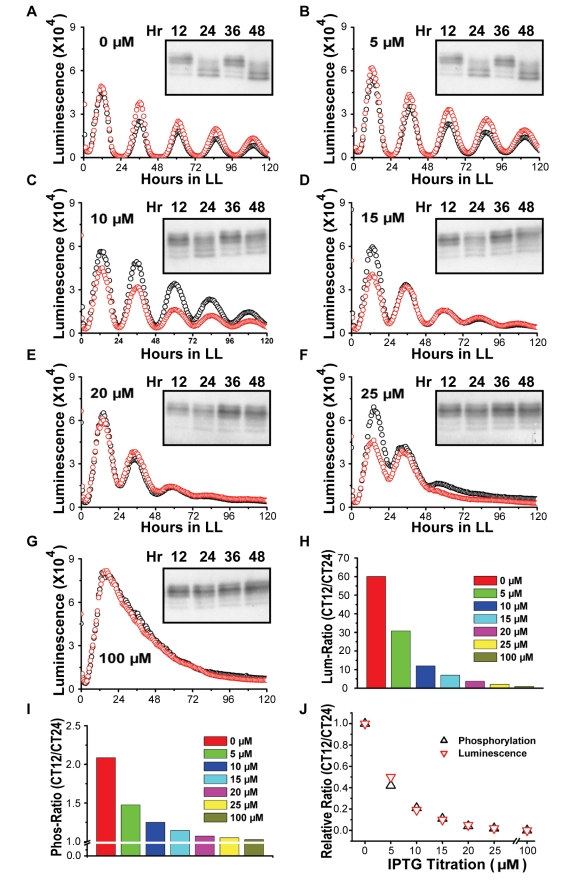
Increasing expression of KaiA suppresses the KaiC phosphorylation rhythm and the gene expression rhythm in parallel. Gene expression was monitored by luminescence from the *kaiBCp*::*luxAB* reporter. The expression level of *kaiA* was under the control of an IPTG-derepressible heterologous *trc* promoter [44]. (A–G) Inducer at the concentration indicated in the upper left corner of each panel (IPTG, from 0 to 100 µM) was added 18 h before the onset of LL to express KaiA. Each panel depicts the effect of that level of KaiA expression on the luminescence rhythm (measurements for 5 d on duplicate samples—black and red circles) and the KaiC phosphorylation rhythm at peak and trough phases for the first 2 d in LL (immunoblot insets to each panel). (H) Amplitude of the luminescence rhythm as a function of KaiA expression level (driven by varying IPTG concentrations). The amplitude was calculated as the ratio of the first luminescence peak to the first trough using the average of the two replicates (background/trough levels were not subtracted). The data in panels A–H made use of the *kaiBC*p*::luxAB* reporter, but similar results were obtained with the *psbAI*p*::luxAB* reporter. (I) Amplitude of the phosphorylation rhythm as a function of KaiA expression level/IPTG. The amplitude was calculated as the ratio of the first peak to the first trough and plotted as a function of [IPTG]. Note ordinal scale break. (J) The ratios of the luminescence and phosphorylation rhythms at 0 µM IPTG were set to 1.0, and ratios of the same two rhythms at 100 µM IPTG were set to 0. These normalized amplitude data were then plotted as a function of IPTG concentration.

First, Kitayama and coworkers over-expressed KaiA under the control of an IPTG-inducible construct and found concentrations of IPTG that apparently suppressed the KaiC phosphorylation rhythm but allowed the rhythm of gene expression (as monitored by a luminescence reporter) to continue [16]. We used the same inducible construct to express KaiA and find in contrast a clear correlation between the suppression of the KaiC phosphorylation rhythm by KaiC hyperphosphorylation and the rhythm of gene expression, as depicted in Figure 2. In response to increasing concentrations of the inducer IPTG, KaiC becomes progressively more hyperphosphorylated (insets to panels A–G of Figure 2), and the luminescence pattern damps to arhythmicity. When the suppression of the phosphorylation rhythm is quantified and normalized (Figure 2I), it correlates precisely with the suppression of the luminescence rhythm (Figure 2H) that reflects the global rhythm of promoter activity (Figure 2J). This precise correlation strongly supports the interpretation that repression of the PTO (as gauged by the KaiC phosphorylation rhythm) leads inevitably to the suppression of the emergent global rhythm of gene expression (Figure 2J). The basis of the discrepancy between our results and those of Kitayama and coworkers is not clear, but it might be explained as a population phenomenon—perhaps there are a few cells in the population with a higher resistance to the IPTG induction that continue to oscillate their KaiC phosphorylation and luminescence in the experiments of Kitayama and coworkers; this could lead to an apparent suppression of KaiC phosphorylation in the population as measured by the relatively insensitive immunoblotting technique, whereas the few rhythmic cells confer a weak, low amplitude rhythm of luminescence (which is a very sensitive gauge). Moreover, simulations with the model that we introduce below indicate that even a very low amplitude rhythm of KaiC phosphorylation in individual cells (that might be undetectable by immunoblotting) can result in a measurable oscillation of transcriptional activity (Figure S5).

**Figure 3:**
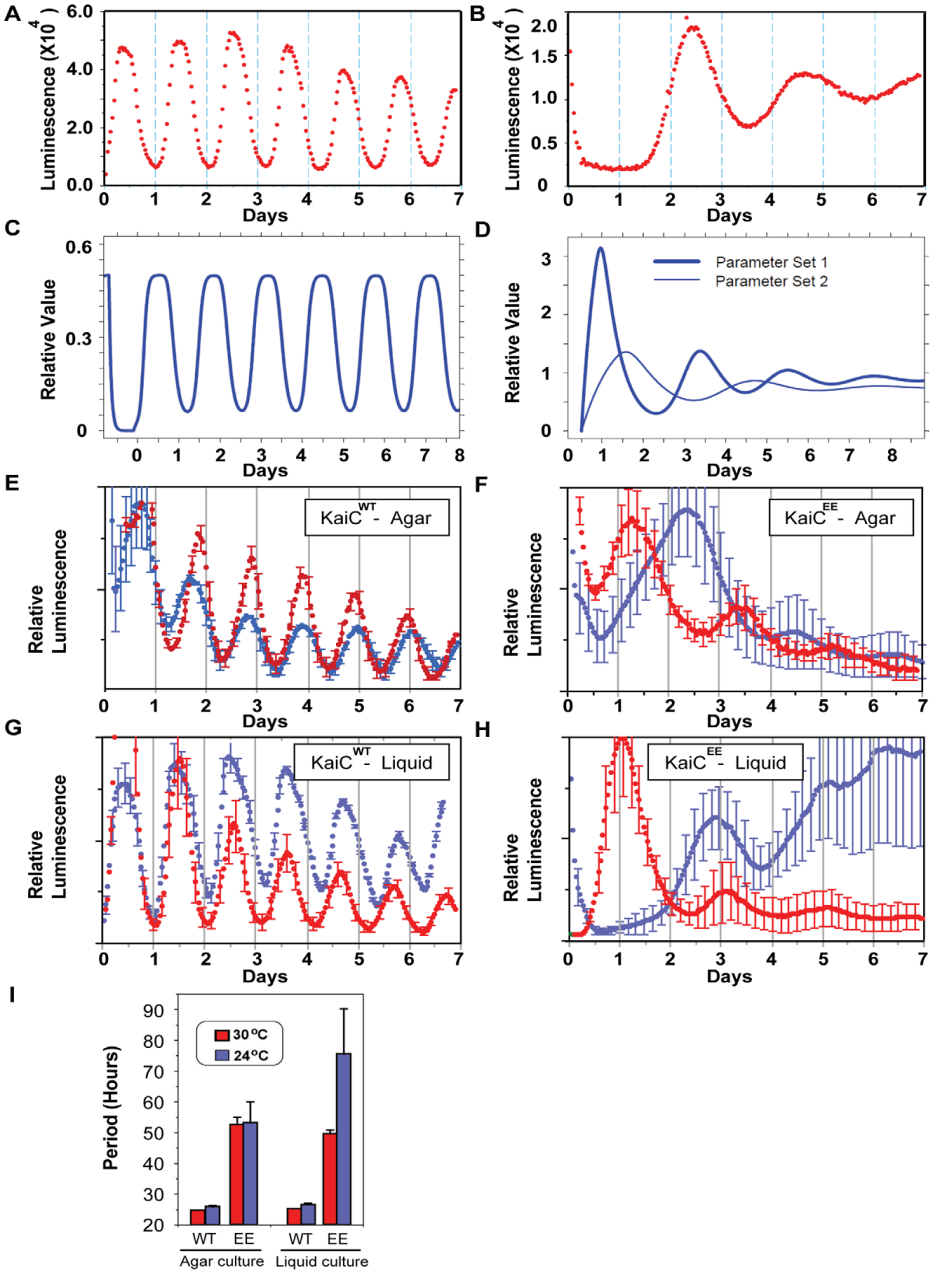
Cells expressing KaiC^EE^ exhibit damped oscillations in LL that have an abnormally long period and are not temperature compensated under different metabolic conditions. (A) Cells expressing KaiC^WT^ show robust circadian oscillations in LL at 30°C with a period of ∼25.4 h. (B) Cells expressing KaiC^EE^ (S431E/T432E) exhibit damped, long-period oscillations in LL at 30°C (period ∼58 h, another example is shown in Figure S6). (C) Simulated circadian oscillatory dynamics of KaiC mRNA abundance in the KaiC^WT^-expressing strain in constant light (LL) in the combined PTO/TTFL model of the KaiABC oscillator. (D) Characteristic simulated oscillatory dynamics of KaiC mRNA in the KaiC^EE^-expressing strain in constant light (LL) using a generic TTFL without a PTO cycle. In the simulations, KaiB•KaiC^EE^ complexes suppress transcription. A constant light-dependent degradation term (proportional to concentration) removes complexes; translation from mRNA creates KaiB and KaiC^EE^. The output of simulations from two different parameter sets of the TTFL is shown; damped oscillations of varying period are typical for a wide range of parameters in the model. Details of the model (differential equations and parameter values) can be found in Text S1. (E–H) Rhythms of luminescence in vivo at 24°C (blue traces) versus 30°C (red traces) under different metabolic conditions: (E) WT cultures on agar medium, (F) KaiC^EE^ cultures on agar medium, (G) WT planktonic cultures in liquid medium, (H) KaiC^EE^ planktonic cultures in liquid medium. (I) Period estimates for the data of panels E–H, where WT = KaiC^WT^ and EE = KaiC^EE^ strains. Error bars in panels E–I are S.D. (*n* values are as follows in panels E–I: 6 for WT at 24°C, 15 for KaiC^EE^ at 24°C, and 5 for both WT and KaiC^EE^ at 30°C). Note that the number of days plotted on the abscissae is different among panels A/B, C/D, and E–H.

**Figure 4:**
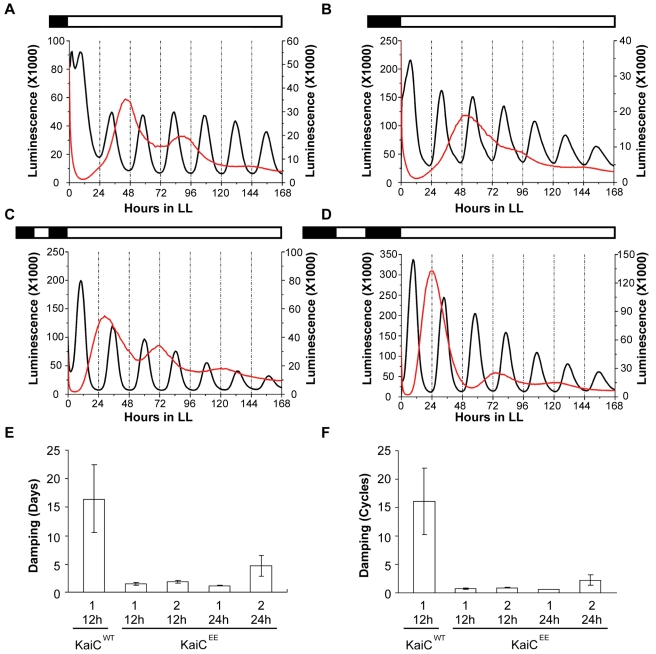
Prior entrainment conditions determine the rate of damping in cells expressing KaiC^EE^. Cells were in LL at 30°C before and after the following entrainment conditions: (A) one 12 h dark pulse, (B) one 24 h dark pulse, (C) two 12 h dark pulses separated by one 12 h light pulse (i.e., 1.5 cycles of LD12:12), and (D) two 24 h dark pulses separated by one 24 h light pulse (i.e., 1.5 cycles of LD24:24). In all panels, the left ordinate is the luminescence level of the WT strain and the right ordinate is the luminescence level of the KaiC^EE^ strain. In panels A–D, the black traces are the WT luminescence and the red traces are the KaiC^EE^ luminescence (these traces are the average of duplicate measurements, see Figure S8 for all raw data). (E) Damping analysis as the number of days required for the amplitude of the rhythm to decrease to 1/e (≈36.79%) of the starting value. (F) Damping analysis as the number of cycles required for the amplitude of the rhythm to decrease to 1/e. In panels E and F, *n* = 5 for KaiC^WT^ and *n* = 7 for each of the KaiC^EE^ sample sets; error bars are SEM.

A second method by which Kitayama and coworkers achieved constitutive hyper-phosphorylation of KaiC was by mutation of the critical phospho-sites on KaiC (S431 and T432) to glutamate residues, thereby creating KaiC^EE^ (i.e., S431E and T432E). KaiC^EE^ is a phosphomimetic of hyper-phosphorylated KaiC that cannot have its phosphorylation status regulated further since sites 431 and 432 are now blocked by the glutamate residues [16]. When the endogenous *kaiC* gene is replaced with a mutated gene encoding KaiC^EE^, there are long-period rhythms of luminescence in vivo at 30°C [16]. We can replicate the results of Kitayama and colleagues with KaiC^EE^, but we interpret those results differently. Our results may be seen most clearly by contrasting Figure 3A with Figure 3B. The KaiC^WT^ strain oscillates robustly for at least 6–12 cycles in LL at 30°C with a period of ∼25.4 h (there is a slight damping over time due to growth/density of cells and depletion of medium, Figure S6A). Conversely, with the KaiC^EE^ strain, not only are the periods much longer (average >50 h) and more variable than for KaiC^WT^ at 30°C, but more crucially the rhythms damp significantly over time (Figures 3B, 4, and S6B). This damping is a consistent feature of the rhythms with the KaiC^EE^ strain at 30°C, and it is also evident in the data of Kitayama and coworkers [16].

Even more interesting, temperature compensation of the KaiC^EE^ strain is not stable under different metabolic conditions. In healthy cultures on agar, colonies of wild-type and KaiC^EE^ strains show temperature compensated rhythms (Figure 3E versus 3F, 3I); when the Q_10_ of these data are calculated (as in [20]), the Q_10_ for cultures on agar was 1.08 for wild-type cells and 1.02 for KaiC^EE^ cells (however, the variability of periods among KaiC^EE^ strains is significantly larger than for wild-type strains, Figure 3I). Conversely, for healthy cultures in liquid medium (“planktonic cultures”), a temperature compensation defect is obvious in the KaiC^EE^ strain (Figure 3G versus 3H, 3I). In particular, planktonic wild-type cells show practically the same period at 24°C and 30°C (Figure 3G, 3I) with a Q_10_ = 1.08. However, planktonic KaiC^EE^ cells show dramatically different periods at 24°C versus 30°C (Figure 3H, 3I; Q_10_ = 2.02) that are drastically more variable at 24°C than for wild-type cells (compare Figure 3G with 3H, and S.D. bars in Figure 3I). Metabolic conditions for bacteria in liquid culture are different from those on agar, and therefore temperature compensation is dependent on metabolic conditions in the KaiC^EE^ strain, but not in wild-type cells. Kitayama and coworkers also showed data for a strain expressing constitutively non-phosphorylated KaiC, named K294H, but we find the rhythms of this strain to be highly unstable with respect to period, phase, and amplitude as shown in Figure S7 and will therefore not be considered further here.

Moreover, the number of cycles exhibited by KaiC^EE^ strains before they damp out is a function of the number of environmental LD cycles experienced by the cells prior to release into LL (Figures 4 and S8). In these experiments, we grew cells expressing either KaiC^WT^ or KaiC^EE^ in LL, then gave them 1–2 cycles of LD with either 12 or 24 h dark intervals, followed by a final release to LL and the monitoring of luminescence. For cultures expressing KaiC^WT^, there were consistent and robust rhythms in the final LL that damped at a slow rate (Figures 4 and S8). On the other hand, cells expressing KaiC^EE^ exhibited obvious damping that was a function of the number of prior cycles of LD—two cycles of LD promoted longer-lasting oscillations than one cycle of LD; this difference between rhythms expressed by KaiC^WT^ versus KaiC^EE^ strains was most obvious with the LD 24:24 conditions (Figure 4E and 4F; compare Figure 4B versus 4D and Figure S8F versus Figure S8H). These data imply that the rhythms expressed by the KaiC^EE^ strain are driven by a damped oscillator whose persistence can be cumulatively stimulated by increasing the number of cycles of driving stimuli.

### Modeling Damped, Long-Period Oscillations in the KaiC^EE^ Strain

Building upon our previous stochastic model of the PTO [13] to simulate the larger circadian system that includes transcription and translation, we constructed a mathematical model where the core PTO oscillator is coupled to a “slave” TTFL oscillator in which the KaiB•KaiC complex nonlinearly suppresses transcription of the *kaiBC* gene (see Methods and Text S1 for a complete model description). This negative feedback is based on generic TTFL repression adapted from a *Drosophila* clock model [21]. To understand how a damped, long-period oscillation might persist in the KaiC^EE^ strain, it is only necessary to consider the TTFL portion of the model. Briefly, starting with low quantities of KaiB•KaiC, repression of *kaiBC* transcription is low and KaiB and KaiC^EE^ protein abundances increase (*kaiB* and *kaiC* genes are adjacent and transcribed as a dicistronic mRNA [3]). KaiB associates with KaiC^EE^ and the level of KaiB•KaiC^EE^ complex increases; this constitutively “hyper-phosphorylated” KaiB•KaiC^EE^ complex acts non-linearly to repress further transcription of *kaiBC* mRNA. As KaiB and KaiC^EE^ protein abundances reach their peak, degradation takes over the dynamics in LL such that KaiB•KaiC^EE^ levels then drop, relieving the suppression of *kaiBC* and typically resulting in oscillatory dynamics. However, as the model shows, because the phosphorylation status of KaiC^EE^ cannot be altered, the transcription and translation loop does not show sustained oscillations (i.e., *kaiBC* mRNA exhibits damped oscillatory dynamics in LL, Figure 3D). On the other hand, inclusion of a PTO with the TTFL for KaiC^WT^ generates robust and consistent oscillations (Figure 3C). In addition, the period of the damped KaiC^EE^ cycles are significantly longer than those of the sustained KaiC^WT^ cycles (compare Figure 3C with 3D). Therefore, a generic TTFL model can accurately reproduce the damped, long-period oscillation of cells expressing KaiC^EE^. We therefore conclude that the data with KaiC^EE^ (Figures 3B, 4, ref. [16]) can be faithfully interpreted in terms of a damped “slave” TTFL oscillator in which a self-sustained PTO pacemaker is embedded.

### Modeling the Larger PTO/TTFL System In Vivo

We used the same TTFL repression function from the KaiC^EE^ model described in the previous section to simulate the in vivo oscillator with KaiC^WT^ consisting of a PTO and TTFL in LL, DD, or LD conditions. The resulting simulations compare changes in protein, mRNA, and phosphorylation levels with experimental data. Briefly (and in simplified form), the PTO portion of the model consists of the following cycle (not including the dissociation kinetics):
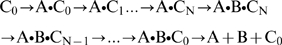
where “C_i_” = KaiC hexamers with “i” number of phosphates, “A” = KaiA, “B” = KaiB, “A•C_0_” is the complex between KaiA and unphosphorylated KaiC, and so on. The exchange of KaiC monomers among the hexamers synchronizes the phosphorylation status within the population of KaiC molecules [11],. In our ordinary differential equation (ODE) model, initially unphosphorylated KaiC binds KaiA and proceeds approximately in a sequence through the phosphorylation states C_1_,C_2_, … C_12_. Hyper-phosphorylated KaiC associates with KaiB, and the KaiB•KaiC complex sequesters KaiA. This sequestration nullifies the stimulatory effect of KaiA and the system dephosphorylates. Synchronization of the phosphorylation status among the KaiC hexamers in the population by KaiC monomer exchange results in sustained oscillations that do not dampen. On the other hand, in the absence of synchronization, this “cyclic” ODE system shows damped oscillations, as we had previously observed with an explicit stochastic matrix model of hexamer interactions [13]. The complete ODE model that comprises the PTO and TTFL includes the association/dissociation kinetics for each state of KaiC with KaiA and KaiB, as well as KaiC auto-phosphorylation and auto-dephosphorylation kinetics. We have not included a more complicated site-dependent (S431 and T432) model of KaiC phosphorylation as experimentally observed [22],[23], resorting instead to a simpler yet effective description of net KaiC phosphorylation level within the population (this PTO model using net KaiC phosphorylation levels and complex association/dissociation kinetics implicitly includes the effects of site-dependence in a phenomenological manner).

**Figure 5:**
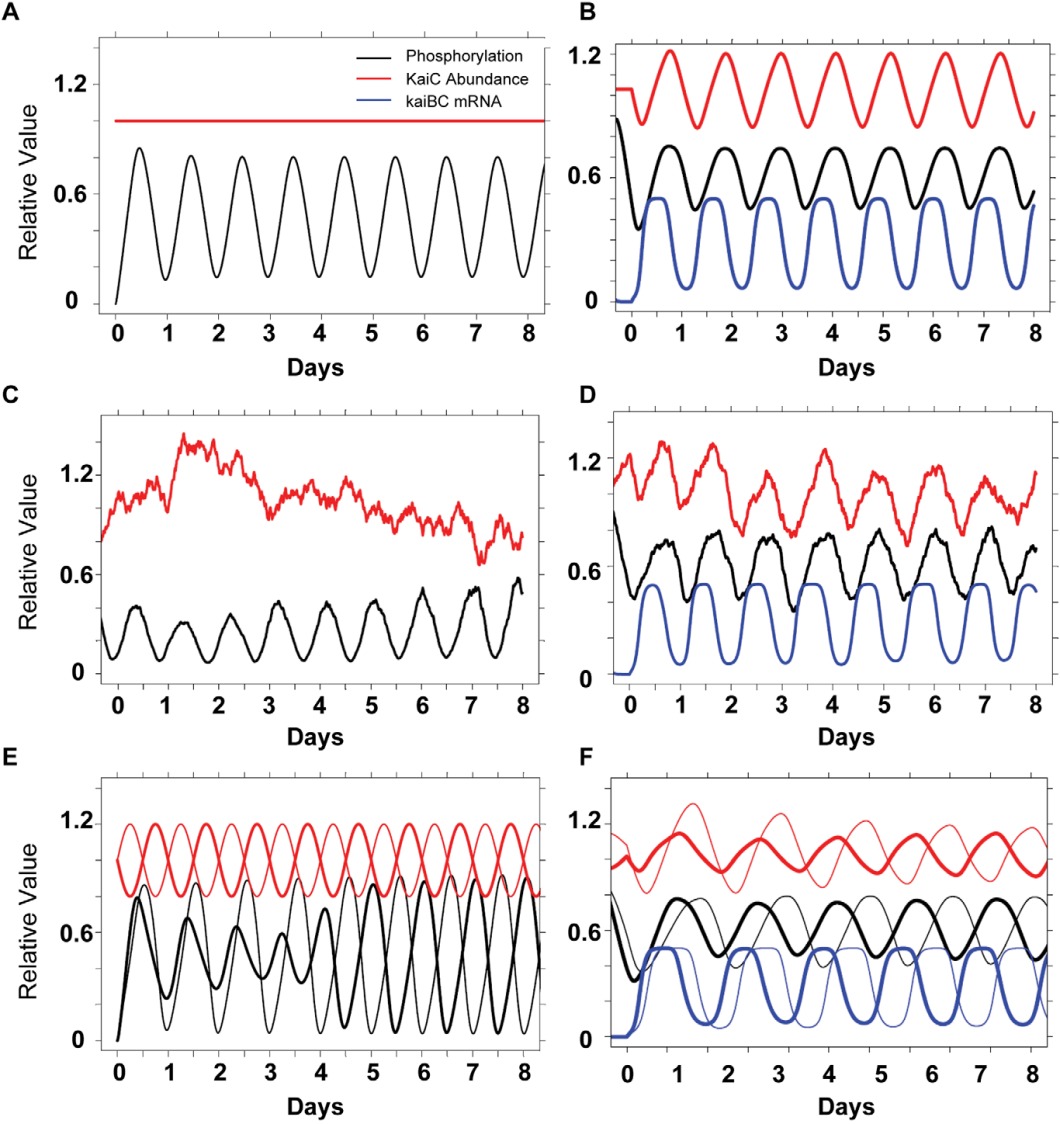
Simulations derived from the PTO/TTFL model, including resilience and phase-locking. (A) PTO alone: In the absence of transcription and translation (i.e., for the in vitro reaction or for cells in DD), KaiC abundance is constant (red trace) and simulation of the PTO model indicates a sustained circadian oscillation in KaiC phosphorylation (black trace). Phosphorylation is reported as a fraction of total KaiC. The initial ratios in simulations are 1∶1.5∶1 KaiA:KaiB:KaiC (dimer, tetramer, hexamer) with a nominal initial KaiC concentration of 1 µM (at initial conditions, KaiC is in the unphosphorylated form). (B) Combined PTO/TTFL: Inclusion of a simple TTFL in which hyperphosphorylated KaiB•KaiC complexes that suppress *kaiBC* transcription show sustained circadian oscillations in KaiC phosphorylation (black), KaiC abundance (red), and *kaiBC* mRNA (blue) in LL. The phase relationships are consistent with previous in vivo studies of the cyanobacterial system. Simulations are shown following two simulated LD 12:12 cycles. KaiC abundance has been scaled to a mean of 1 (max value ∼3.5 KaiC_0_). Levels of *kaiBC* mRNA are reported as a fraction of initially unphosphorylated KaiC ( = KaiC_0_). (C) PTO resilience as assessed by the effect of noisy unphosphorylated KaiC on the PTO. Random noise in unphosphorylated KaiC was introduced as shown (red trace). KaiC abundance is normalized to a mean of 1.0 (initial concentration is set at 1 µM). The phosphorylation rhythm (black) remains robustly rhythmic despite significant fluctuations in KaiC abundance. (D) Combined PTO/TTFL resilience as assessed by the effect of noisy unphosphorylated KaiC on the PTO/TTFL system. Random noise in unphosphorylated KaiC was introduced at the same level as in Panel C. However, with the inclusion of the TTFL, the same noise fluctuations (as in panel C) result in noisy KaiC abundance oscillations (red trace; KaiC abundance is normalized to a mean of 1.0). The phosphorylation rhythm (black) remains circadian despite significant fluctuations in KaiC but is perturbed by fluctuations in abundance. The abundance of *kaiBC* mRNA (blue trace) is much less noisy as it reflects the effect of hyper-phosphorylated KaiB•KaiC complexes. Monomer exchange reactions in the PTO decrease the effect of noisy fluctuations in unphosphorylated KaiC fluctuations on the hyper-phosphorylated states. (E) Phase-locking in the PTO. External circadian sinusoidal driving of the abundance of unphosphorylated KaiC in two different phases (0 h, thick lines and 12 h, thin lines) results in the same ultimate asymptotic phase relationship between abundance (red) and phosphorylation (black). At the beginning of the simulation, the phase relationship between KaiC phosphorylation and KaiC abundance is optimal for the thin-trace case and remains so. However, for the thick-trace case, the initial conditions have a non-optimal phase between KaiC phosphorylation and KaiC abundance that resolves into the optimal relationship after about 4 d. (F) Phase-locking in the combined PTO/TTFL. External circadian sinusoidal driving of the abundance of unphosphorylated KaiC as in Panel E in two different phases (0 h, thick lines and 12 h, thin lines) results in the same asymptotic phase relationship between abundance (red), phosphorylation (black), and *kaiBC* mRNA (blue). The result is similar to that simulated in Panel E except that a final effect on mRNA levels is shown. Details of the models (differential equations and parameter values) can be found in Text S1.

We modeled the TTFL using a generic nonlinear repression term (as in the KaiC^EE^ case described in the previous section) and a light-dependent protein degradation term (based on the data of Figure S3). For the KaiC^WT^ strain, the PTO shows robust sustained circadian oscillations in net phosphorylation level (Figure 5A) for a variety of parameter choices. Inclusion of a TTFL without any specific parameter “tuning” results in a circadian oscillation in KaiB/KaiC protein abundances, *kaiBC* mRNA levels, and KaiC phosphorylation status in LL (Figure 5B). In DD when transcription/translation and protein degradation is turned off (Figure S3 and [6]), there is a circadian KaiC phosphorylation rhythm due solely to the PTO (Figure 5A). Therefore, this model that combines a core PTO plus a damped “slave” TTFL oscillator can accurately reproduce the sustained circa-24 h rhythms of protein/mRNA abundances and KaiC phosphorylation in the KaiC^WT^ strain, while the TTFL portion alone can create damped, long-period oscillations of *kaiBC* mRNA as observed in cells expressing KaiC^EE^ (Figures 3C, 3D, 5A, and 5B).

### Resilience of the PTO/TTFL Model to Noise

As further evidence that the PTO can function as the core circadian pacemaker in the larger system, we included noisy fluctuations in the concentration of unphosphorylated KaiC in the PTO/TTFL model. For the PTO alone, both (i) the stochastic matrix model for small molecular numbers (unpublished data) and (ii) the ODE model allow robust circadian oscillations in the presence of noisy fluctuations of unphosphorylated KaiC levels (Figure 5C). When the TTFL is included, the simulated system continues to show robust circadian oscillations in the phosphorylation rhythm and circadian dynamics for the protein abundance even though the same external noise fluctuations were introduced as in the PTO simulation (Figure 5D). These modeling data are supported by experimental data in the LD 2:2 cycle—the 4 h light/dark cycle drives a 4 h modulation of photosynthesis and metabolism, leading to a noisy KaiC abundance pattern (Figure 1E); nevertheless, KaiC phospho-status exhibits a clean circadian rhythm (Figure 1F) as does the luminescence indicator of transcriptional activity (Figure S1C).

**Figure 6:**
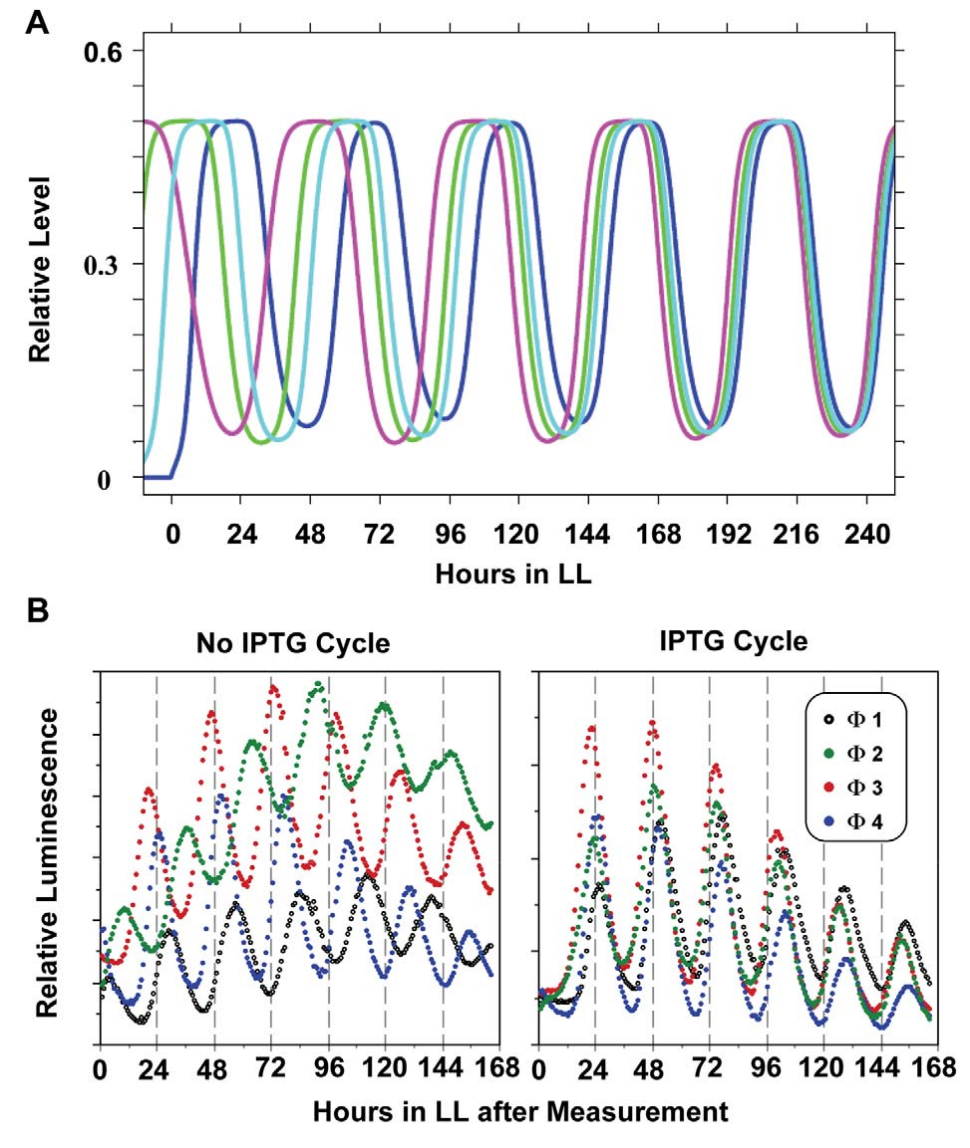
Experimental test of phase locking and entrainment. (A) Model predictions: simulated phase-locking of *kaiBC* mRNA rhythms in the combined PTO/TTFL model for four different starting initial phases of mRNA abundance rhythms. Sinusoidal external driving (of unphosphorylated KaiC protein) is implemented as in Figure 5E and 5F. The four phases preferentially lock into a single phase set by the external driving rhythm. The blue trace illustrates the case of initial conditions that are already in the optimal phase relationship to the driving rhythm. (B) Experimental confirmation: cycles of induction of new KaiC synthesis cause phase-locking. Entrainment to four different LD12:12 cycles in which the phase was set to times 0, 6, 12, 18 (i.e., at 6 h intervals) prior to LL release generates four separate populations of cells that are roughly different in phase by 6 h. Cells in four separately phased populations were then treated with two cycles of 0: 0 µM IPTG (i.e. no IPTG cycle, left panel) or 0: 5 µM IPTG (i.e. IPTG cycle: 12 h IPTG, 12 h no IPTG, 12 h IPTG, right panel), and released to free-run in LL. See Figure S9A for an illustration of the experimental protocol.

These simulations of noisy KaiC abundance examine typical fluctuations that may occur in cellular components due to intrinsic noise in transcriptional and translational processes, cell division, and external random perturbations on the clock components. We also simulated the effect of non-random perturbations of abundance on the system both for the PTO alone and including the TTFL. For example, experimental manipulation of KaiC abundance as pulsative increases in KaiC levels has been reported to reset the phase of the circadian system in vivo [3],[4]. To examine the effects of non-random KaiC perturbations, we first introduced a sinusoidally driven rhythm of KaiC protein abundance to determine how the PTO would respond. The PTO's phosphorylation rhythm was begun in different initial phases relative to the driving KaiC sinusoidal abundance (Figure 5E). After a few “sorting out” cycles, the PTO system shows “phase locking” to the abundance rhythm in a specific phase relationship. The intuitive reason for this effect is that the KaiC phosphorylation rhythm is optimal only when synthesis of unphosphorylated KaiC occurs in phases near the trough of the phosphorylation rhythm (see below). When the TTFL is included, the effect of driving the KaiC abundance externally results in an analogous phase-locking effect (Figures 5F, 6A).

### Experimental Tests of Model's Prediction for Phase Locking

One experimental test of the model's prediction can be provided by *kaiBC* transcription and translation under the control of an oppositely phased promoter—the *purF* promoter [24]. The cyanobacterial clock system under directed anti-phase expression of the *kai* genes was reported to have phase relationships that are practically the same as wild-type [24], and we have confirmed those results (unpublished data). Those results support the model's prediction of “locking in” to the preferred phase relationship between the KaiC phosphorylation rhythm and synthesis of unphosphorylated KaiC.

Another experimental test of the model's prediction is to experimentally create a cycle of new KaiC synthesis (of necessarily unphosphorylated KaiC) in vivo that begins in different initial phase relationships to the rhythm of KaiC phosphorylation. As shown in Figure 6A (and Figure 5F), these varying phase relationships should resolve after a few cycles into a single steady-state phase relationship between new KaiC synthesis and the KaiC phosphorylation rhythm. Using a strain with additional KaiC expression driven by an IPTG-inducible promoter (*trc*p) at an ectopic site in the chromosome, we experimentally created a 24 h cycle (12:12) of new KaiC synthesis within a physiological range of KaiC abundance by administering two cycles of 5 µM IPTG (12 h IPTG, 12 h no-IPTG, 12 h IPTG, then a free-run without IPTG; Figure S9A). This concentration of IPTG will increase KaiC levels by 40%–50% over basal values and it concomitantly decreases the phosphorylation status of KaiC (Figure S9B–D). Four cultures that had been phased into four distinct phases (0, 6, 12, and 18 h apart) were treated with this IPTG:no-IPTG cycle. As shown in Figure 6B, we obtained the clear result that two cycles of new KaiC synthesis caused a locking of the four cultures to a single synchronous phase. Of particular significance is that the phase relationship of these synchronized luminescence rhythms to the cycle of new KaiC synthesis was as predicted by the model. Therefore, the core biochemical PTO is not totally insensitive to changes in levels of unphosphorylated KaiC, but it can be entrained by cycles of KaiC that result from the damped TTFL.

## Discussion

### A PTO Pacemaker Embedded within a TTFL Slave Oscillator

**Figure 7:**
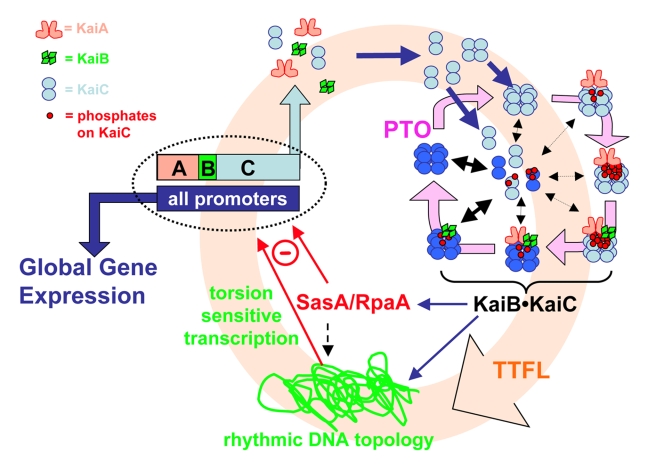
The core PTO is embedded in a larger TTFL. The PTO is linked to the damped TTFL (indicated by the pink background circle) by transcription and translation of the *kaiABC* cluster. Global gene expression is mediated by rhythmic modulation of the activity of all promoters, including those driving the expression of the central clock gene cluster, *kaiABC* ( = ABC in figure). Rhythmic DNA torsion and/or transcriptional factor activity (e.g., RpaA/SasA) modulate global promoter activities. Cyclic changes in the phosphorylation status of KaiC that mediate the formation of the KaiB•KaiC complex regulate DNA topology/transcriptional factors. The PTO (cycle connected by lavender arrows in upper right quadrant) is determined by KaiC phosphorylation as regulated by interactions with KaiA and KaiB. Robustness is maintained by synchronization of KaiC hexameric status via monomer exchange [11],[13]. Monomer exchange is depicted in the figure by “dumbbell” KaiC monomers exchanging with KaiC hexamers in the middle of the PTO cycle; phase-dependent rate of monomer exchange is indicated by the thickness of the double-headed black arrows. The shade of KaiC hexamers (dark versus light blue) denotes conformational changes that roughly equate to kinase versus phosphatase forms. New synthesis of KaiC feeds into the KaiABC oscillator as non-phosphorylated hexamers or as monomers that exchange into pre-existing hexamers. If the new synthesis of KaiC occurs at a phase when hexamers are predominantly hypo-phosphorylated, the oscillation of KaiC phosphorylation is reinforced (enhanced amplitude). If on the other hand, new synthesis of unphosphorylated KaiC happens at a phase when hexamers are predominantly hyper-phosphorylated, this leads to an overall decrease in the KaiC phosphorylation status, thereby altering the phase of the KaiABC oscillator (phase shift) and/or reducing its amplitude.

This investigation is the first to our knowledge to investigate the dynamics of a circadian clock in which a PTO is coupled to a damped TTFL. Our data strongly support the interpretation that when the cyanobacterial PTO is suppressed, the emergent circadian rhythms, including the TTFL and global gene expression, are concomitantly suppressed. Moreover, when the PTO is suppressed—either by KaiA-overexpression or by mutation of KaiC—the remaining TTFL shows clear characteristics of a damped oscillator that is effectively a “slave” of the self-sustained PTO. As shown in Figure 7, our model proposes that the PTO is embedded within a TTFL with the KaiB•KaiC complex repressing transcriptional activity through control of chromosomal topology [25] and/or transcriptional factors such as RpaA [15]. This pervasive transcriptional activity regulates global gene expression but also rhythmically regulates new synthesis of KaiA, KaiB, and KaiC, thus completing a transcription and translation loop (Figure 7).

Despite the fact that the PTO can oscillate independently of the TTFL in vitro and in vivo [6],[10], the PTO is not totally independent from the TTFL in vivo. The data and simulations depicted in Figure 5C and 5D imply that stochastic changes in KaiC abundance will not eliminate the circadian dynamics for physiological perturbations. However, experimental manipulations of KaiC abundance as pulsative increases in KaiC levels have been reported to reset the phase of the circadian system in vivo [3],[4]. How can these disparate conclusions be reconciled? A likely answer is that because the newly synthesized KaiC monomers/hexamers are necessarily unphosphorylated, the phase at which they are added into the PTO is critical. If they are added at a phase when most of the KaiC hexamers are phosphorylated, then the newly synthesized proteins will monomer-exchange into the existing population of KaiC hexamers [11]–[13] and alter the phospho-status of the KaiC population, potentially disrupting the PTO. On the other hand, if the newly synthesized and unphosphorylated KaiC proteins are added at a phase when the KaiC population is largely unphosphorylated, then the PTO will not be disrupted and will even be potentially reinforced. Therefore, regular cycles of new KaiC synthesis can entrain. On the other hand, if the KaiC abundance fluctuates randomly with a relatively low amplitude, the system is resilient and its circadian nature dominates. This interpretation is supported by both the experimental data and by the modeling simulations. When KaiC abundance is constant, its phosphorylation status oscillates with a circadian period (experimental data in [6]; simulations: Figure 5A). When KaiC abundance is allowed (or forced) to oscillate, then the PTO locks into a preferred phase relationship to the KaiC synthesis rhythm (resulting from the TTFL) such that newly synthesized KaiC is introduced at phases of the PTO when KaiC is relatively hypo-phosphorylated (experimental data: Figure 6B; simulations: Figures 5E, 5F, and 6A). When the synthesis of KaiC is driven at an unusual phase by an antiphase promoter, the PTO and TTFL nevertheless lock-in at the preferred phase relationship (experimental data in [24] and our unpublished results; simulations: Figure 5F).

What about entrainment to the environmental cycles? Because transcription and translation proceed in the daytime but are turned off in darkness in photoautrophic *S. elongatus* cells [6], there is a daily rhythm of synthesis of the Kai proteins in LD (for the case in LL, see [19]). Therefore, it is reasonable to suppose that daily rhythms of total Kai protein abundance could be an entraining stimulus. However, we did not find such a rhythm of total KaiC abundance in LD 12:12 (Figure 1C). At the same time, we found that degradation of KaiC is minimal in darkness (Figure S3), leading to the interpretation that KaiC synthesis and degradation proceed during the illuminated day phase but essentially counterbalance each other so that there is not a major change in the net KaiC abundance during the day. Then at night, KaiC's degradation is minimal (Figure S3) and its transcription and translation is turned off [6], resulting in a practically constant level of KaiC abundance over the LD 12:12 cycle (Figure 1C). Therefore, it seems unlikely that changes in Kai protein abundance per se in an LD cycle could provide an entrainment mechanism. However, even though total KaiC *abundance* does not oscillate in LD 12:12, new *synthesis* of KaiC does oscillate in LD (on in the day, off at night). Because new synthesis of KaiC provides unphosphorylated protein and could thereby affect the ratio of hyper- to hypo-phosphorylated KaiC (Figure S9D), this stimulus could contribute to the transduction pathway for entrainment. As shown in Figures 5E, 5F, and 6A, simulations predict such a phasing effect, and our experimental test of this prediction by providing a low amplitude rhythm of new KaiC synthesis supports this hypothesis (Figure 6B). In this sense, the TTFL could play a role in both input and output pathways of the PTO pacemaker, as has been suggested for the mammalian clock [26].

### Implications of a PTO Embedded within a Larger TTFL: Robustness

A system composed of a biochemical pacemaker embedded within a damped transcription/translation loop has important implications. Biochemical reactions that involve small numbers of molecules are intrinsically noisy, being dominated by large concentration fluctuations [27],[28]. In general, the number of transcription factor molecules in a prokaryotic cell is small and this could lead to a high intrinsic noise [29]. On the other hand, a PTO that is rooted in the phosphorylation status of thousands of molecules would be expected to be robust in the face of noise. In the case of KaiC, the current estimate is that there are approximately 10,000 KaiC monomers per cell [30]. The model of the PTO supports the hypothesis that the KaiABC PTO is resilient to noise. Figure 5C shows a simulation of noise in the PTO model by introducing fluctuations in the abundance of KaiC in a population of hexamers, and Figure 5D shows the same influence in the combined PTO/TTFL model. Despite the noisy KaiC abundance fluctuations, the circadian rhythm of KaiC phospho-status oscillates consistently, whether the PTO is considered separately (Figure 5C) or within the larger TTFL system (Figure 5D). These modeling data are supported by experimental data in the LD 2:2 cycle—the 4 h LD cycle drives an ultradian modulation of metabolism, leading to a noisy KaiC abundance pattern (Figure 1E); nevertheless, KaiC phospho-status exhibits a clean circadian rhythm (Figure 1F).

Not only is the circadian system resilient within the cell, but it is robust among a population of cells. The experimental observation of reproducible rhythms among non-communicating cyanobacterial cells in populations [31] implies that the ODE model is applicable to modeling the mean population behavior of cells, with the noise reflecting population variance in LD (Figure 5D). Resilience of the daily timekeeper is particularly important for cells that must keep accurate track of time in the face of cell division, when a TTFL might become perturbed because the ratio of DNA to transcriptional factors can change during replication and when DNA can become less accessible during chromosomal condensation in preparation for division. In bacterial oscillators that were experimentally designed to be strict TTFLs, cell division clearly disrupts the phasing and/or period of these synthetic clocks [32]. Cell division or chromosomal events should not, however, perturb a strictly biochemical oscillator, as observed in *S. elongatus* [31],[33]. Therefore, evolution appears to have selected in cyanobacteria a core biochemical pacemaker that regulates a TTFL that in turn regulates global DNA topology and gene expression.

### Implications of a PTO Embedded within a Larger TTFL for Eukaryotic Circadian Clocks

Early evidence for a TTFL as the core pacemaker in the cyanobacterial system came from numerous studies that showed the same phenomena which has been used to support a TTFL model in eukaryotes, namely: (1) “clock genes” deemed to be essential based on knockout studies [3], (2) rhythmic abundances of mRNAs and proteins encoded by clock genes [3],[4],[6], (3) autoregulatory negative feedback of clock proteins on their gene's transcription [3],[7], and (4) phase setting by pulsatile expression of clock genes [3],[4]. Eukaryotic circadian genes have no detectable homology to *kaiABC* sequences, so if there is an evolutionary relationship between the bacterial and eukaryotic systems, it is so diverged as to be undetectable by genetic sequence comparisons. But how about the possibility of convergent evolution to a fundamentally similar biochemical mechanism? Could self-sustained biochemical core oscillators underlie eukaryotic clocks? It might seem implausible that independent origins for clocks would converge upon an essentially similar core PTO made more robust by an overlying TTFL. However, the advantages that accrue to the cyanobacterial system by having a post-translational mechanism at its core are also relevant to eukaryotic clocks [5]. For example, individual mammalian fibroblasts express cell-autonomous, self-sustained circadian oscillations of gene expression that are largely unperturbed by cell division [34],[35] in a fashion reminiscent of cyanobacteria [31],[33]. In contrast, synthetic TTFL oscillators constructed in mammalian cells (CHO cells) only display reproducible oscillations when the cells are arrested in G1 phase of the cell cycle by cultivating the cells at 30°C ([36], Dr. Marcel Tigges, personal communication).

Could the imperturbability of circadian clocks even when buffeted by the gusts of metabolic changes provoked by cell division provide an evolutionary driving force for clock mechanisms in disparate organisms to converge on a relatively similar core mechanism? Perhaps. Recent results from the mammalian circadian clock do not easily fit into the original TTFL formulation. For example, mammalian clocks are surprisingly resilient to large changes in transcriptional rate [37], but tight regulation of transcriptional rate would be expected to be necessary if it is a state parameter in a TTFL clock. Also, the mammalian clock is resilient to clamping the level of some of the mammalian clock proteins whose cycling had been thought to be essential [26],[38]. Moreover, recent results have led to a greater appreciation of the role of small signaling molecules in the mammalian clock [39]. Finally, classical experiments in eukaryotic algae have shown that persisting circadian rhythms are possible in enucleated cells (in *Acetabularia* [40],[41]) or under translational control in the absence of transcription (in *Gonyaulax* [42]). Are *Acetabularia* and *Gonyaulax* anomalous cases, or are they relevant indicators of the underlying capabilities of the eukaryotic clockwork? Our growing appreciation of the cyanobacterial system combined with results from eukaryotic clocks that are inconsistent with a sustained TTFL pacemaker embolden such speculations [26],[39],[43]. At the least, the studies on prokaryotic cyanobacteria lead to more rigorous criteria for distinguishing whether a TTFL is at the core of eukaryotic clocks.

## Materials and Methods

See Text S1 for details of experimental procedures and mathematical model description.

### Strains, Culture Conditions, and Luminescence Assay of In Vivo Rhythms

The cyanobacterium *Synechococcus elongatus* PCC 7942 was transformed with a luciferase reporter of either the *psbAI* promoter (*psbAIp::luxAB*) or the *kaiB* promoter (*kaiBCp::luxAB*). Luminescence rhythms from the *psbAIp::luxAB* or *kaiBCp::luxAB* reporters are approximately equivalent in phase and intensity. For experimental expression of KaiA or KaiC, the *kaiA* or *kaiC* genes were fused to an IPTG-derepressible heterologous *trc* promoter (*trc*p) to make *trc*p*::kaiA* or *trc*p*::kaiC* expression strains (in neutral site II, NSII). The KaiC^EE^ strain has the wild-type *kaiC* gene (*kaiC^WT^*) replaced with a double mutant KaiC^S431E/T432E^ [20]. For measurement of KaiC degradation rate, a *kaiC*-null strain was transformed with *trc*p*::kaiC^WT^* in NS II [7]. All strains were grown in BG-11 medium and experiments were performed at 30°C except as indicated in Figure 3.

For assay of in vivo rhythms of *psbAI* or *kaiBC* promoter activity, luminescence emitted by the *psbAIp::luxAB* or *kaiBCp::luxAB* reporter was assayed as previously described from liquid cultures [4] or from colonies on agar [7]. Cells were grown in constant light (LL; cool-white fluorescence at 40–50 µE/m^2^s), given 1∼2 light:dark cycles (e.g. LD 12:12) to synchronize the cells in the population, and finally released into LL for assay of free-running luminescence rhythms. Analysis of damping was performed with the LumiCycle data analysis program (Actimetrics, Evanston, IL, courtesy of Dr. David Ferster) as described in Text S1. Damping rate (d) is the number of days required for the amplitude of the rhythm to decrease to 1/e (≈36.79%) of the starting value.

### KaiC Abundance and Phosphorylation Rhythms In Vivo; KaiC Phosphorylation Rhythms In Vitro

For the experiments of Figure 1, *S. elongatus* cells were harvested every 4 h for the LL and LD12:12 conditions and every 1 h for the LD2:2 conditions. Total protein was extracted and equal amounts of proteins were loaded into each well for SDS-PAGE and immunoblotting [4]. KaiC protein abundance was determined on 15% SDS-PAGE gels (to obtain a single KaiC band), whereas KaiC phosphorylation was determined on 10% SDS-PAGE gels (to separate the various KaiC phosphoforms). For assay of KaiC phosphorylation rhythms in vitro, Kai proteins from *S. elongatus* were expressed in *Escherichia coli* and purified as described previously [13]. In vitro reactions were carried out at 30°C using standard Kai protein concentrations: 50 ng/µl KaiA, 50 ng/µl KaiB, and 200 ng/µl KaiC as described previously [13].

### Phase Locking by Cycles of Low Levels of KaiC Expression

Cells harboring the *trc*p*::kaiC* expression construct were inoculated onto nitrocellulose membranes that were placed on the surface of BG-11 agar plates. After 5 d of growth in LL, these cultured membrane plates were divided into four groups and entrained with two LD12:12 cycles (Figure S9A). There were a total of 4 different phasings of the LD12:12 cycles (Φ1, Φ2, Φ3, and Φ4) that were different from each other by 6 h (i.e., starting at laboratory clock time 00:00, 06:00, 12:00, and 18:00). After the final dark interval was completed for the last group of plates (Φ4), the cultures were transferred back and forth between pre-warmed fresh BG-11 agar plates containing 0 or 5 µM IPTG for two IPTG cycles as follows: 12 h IPTG ( = 5 µM IPTG) followed by 12 h no-IPTG ( = 0 µM IPTG) followed by 12 h IPTG ( = 5 µM IPTG), as depicted in Figure S9A. This protocol created an experimentally controlled 12 h:12 h cycle of new KaiC synthesis (the parallel control cultures were transferred every 12 h among no-IPTG plates). We determined that a concentration of 5 µM IPTG increases KaiC abundance within cells by only ∼40%–50% above basal levels (Figure S9C). After the final IPTG cycle, measurement of the luminescence rhythms in the cultures from each of these phases was performed in LL as described above.

### Modeling

The model was implemented in Fortran (G77) using a fourth order Runge-Kutta routine for solving the coupled set of ODEs. The TTFL portion of the combined PTO/TTFL model is modified from the model of Goldbeter [21]. See Text S1 for model description and the parameters used in the figures.

## Supporting Information

Figure S1 **Representative immunoblots for KaiC abundance (A) and KaiC phosphorylation (B) in LL, LD12:12, and LD2:2.** Densitometry of these blots is shown in Figure 1. (C) Persistence of 24 h circadian rhythm of luminescence in LD2:2 conditions. Upper panel, growth curve for cells in LD2:2. Lower panel, samples taken from the middle of the light interval or the middle of the dark interval in LD2:2 show robust luminescence rhythms. Before release into LD2:2 conditions, cells were given two LD12:12 cycles. To measure the luminescence of cultures from the batch flasks under LD2:2 conditions, 1 ml of cell culture was manually removed in either the middle of the light portion of LD2:2 or the middle of the dark portion of LD2:2 and transferred to a 20 ml vial with a tube containing *n*-decanal to measure the luciferase activity using a luminometer (Femtomaster FB12, Zylux Corporation, Knoxville, TN, USA). The maximum luminescence level at each time point was plotted for both the middle light (solid square) and the middle dark (solid triangle) collection times.
(0.63 MB PDF)

Figure S2 **Absence of clear 24 h rhythmicity of abundances of KaiA, KaiB, and KaiC in LD12:12 despite the presence of a robust KaiC phosphorylation rhythm.** A representative example is shown of an experiment where the phosphorylation rhythm was robust in LD12:12, while the abundances of the Kai proteins were not clearly rhythmic. Abundance data were collected from immunoblots run on 15% SDS-PAGE gels (to obtain a single protein band), whereas KaiC phosphorylation was determined on 10% SDS-PAGE gels (to separate the various KaiC phosphoforms)[4],[7],[20].
(0.18 MB PDF)

Figure S3 **Degradation of KaiC protein is dependent upon light.** (A) KaiC^WT^ expression was induced in the KaiC^OX^ strain by 100 µM IPTG for 6 h, and then the inducer was washed out and the cells were placed in either LL or DD. Samples were collected at the times indicated and processed for SDS-PAGE and immunoblotting as described previously for this type of degradation assay [7]. (B) Same as in panel A except with KaiC^EE^. (C, D) Quantification of the immunoblot data in panels A and B by Image J, which shows that the degradation of KaiC^WT^ (panel C) and KaiC^EE^ (panel D) in the cells proceeds in LL but is strongly inhibited in DD.
(0.26 MB PDF)

Figure S4 **Elevated levels of KaiA in the in vitro reaction suppress the amplitude of the in vitro phosphorylation rhythm.** KaiA, KaiB, and KaiC were mixed together at the final concentrations described in the Materials and Methods of Text S1 and were then dialyzed against medium without ATP for 24 h. The subsequent addition of 1 mM ATP initiates the in vitro rhythms. 1× KaiA = 50 ng/µl KaiA (duplicate reactions shown in red and black), 2× = 100 ng/µl KaiA (blue), and 4× = 200 ng/µl KaiA (purple). Elevated levels of KaiA cause hyper-phosphorylation of KaiA and suppression of the in vitro phosphorylation rhythm in the hyper-phosphorylated state.
(0.05 MB PDF)

Figure S5 **A simulated low amplitude KaiC phosphorylation rhythm (thick black trace) can be amplified into a larger amplitude rhythm in** ***kaiBC*** **mRNA (thick blue line) and Kai C protein (thick red line) abundance.** The thin lines indicate the control simulation for the TTFL (Figure 5B in main text).
(0.06 MB PDF)

Figure S6 **Another example of the rhythms expressed by KaiC^WT^ (panel A) versus KaiC^EE^ (panel B) strains at 30°C that illustrates the obvious damping of the KaiC^EE^ strain.**
(0.05 MB PDF)

Figure S7 **Strains expressing KaiC^K294H^ are unstable with respect to phase, amplitude, and period.** Upper panel is wild-type, and lower panel depicts a simultaneous recording of luminescence emitted by cells expressing KaiC^K294H^ (KaiC^K294H^ was constructed and expressed as in [16]).
(0.04 MB PDF)

Figure S8 **Raw data for** **Figure 4** **(“Prior entrainment conditions determine the rate of damping in cells expressing KaiC^EE^”).** Luminescence rhythms measured in vivo are shown for the WT strain (panels A–D) and for the KaiC^EE^ strain (panels E–H). Cells were in LL at 30°C before and after the following entrainment conditions: (A, E) One 12 h dark pulse; (B, F) one 24 h dark pulse; (C, G) two 12 h dark pulses separated by one 12 h light pulse (i.e., 1.5 cycles of LD12:12); and (D, H) two 24 h dark pulses separated by one 24 h light pulse (i.e., 1.5 cycles of LD24:24). Each differently colored trace is from an independent measurement; *n* = 5 for each of the KaiC^WT^ sample sets and *n* = 7 for each of the KaiC^EE^ sample sets.
(0.21 MB PDF)

Figure S9 **(A) Protocol of the phase locking experiment depicted in** **Figure 6B** **.** Four separate cultures of the KaiC^OX^ strain were phased to four different phases (Φ1, Φ2, Φ3, and Φ4) by exposure to 12 h dark pulses that were phased 6 h apart (at clock times 00:00, 06:00, 12:00, and 18:00). For the “Controls” cultures, no IPTG cycle was given prior to release into LL and initiation of the measurement of luminescence rhythms at time 0. The experimental “KaiC cycles” samples were exposed to two 12 h administrations of 5 µM IPTG, separated by a 12 h interval of medium without IPTG, thereby creating two 12:12 cycles of IPTG:no-IPTG. (B) Induction of new KaiC synthesis in strain KaiC^OX^ by various concentrations of IPTG leads to changes in KaiC phosphorylation status and abundance. An immunoblot of KaiC is shown in panel B. (C) Densitometry of total KaiC abundance in the blot depicted in panel B. (D) Ratio of hyper-phosphorylated to total KaiC in the blot depicted in panel B. Data were analyzed by Image J.
(0.31 MB PDF)

Text S1 **Text S1 includes a complete description of the mathematical modeling, supplemental methods, and references for the Supporting Information.**
(0.11 MB DOC)

## Abbreviations

ODE: ordinary differential equation
PTO: post-translational oscillator
TTFL: transcriptional/translational feedback loop
