# Supplementary figures and images for: Coupling of a Core Post-Translational Pacemaker to a Slave Transcription/Translation Feedback Loop in a Circadian System
Source: PLoS Biol. 2010 Jun 15;8(6):e1000394. doi: 10.1371/journal.pbio.1000394 (PMC2885980; doi:10.1371/journal.pbio.1000394)

Fig. S1

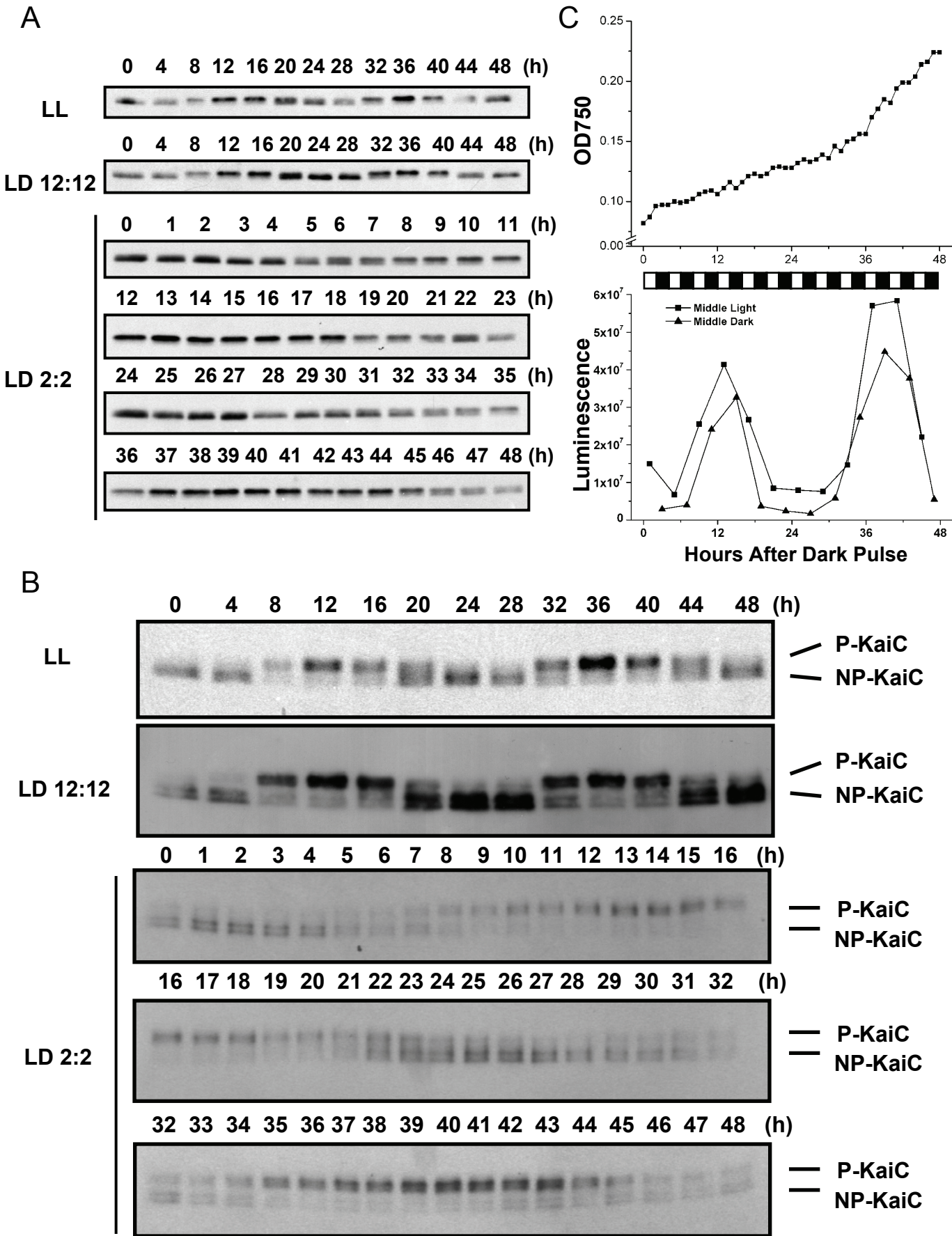

Supplement: Figure S1 — Representative immunoblots for KaiC abundance (A) and KaiC phosphorylation (B) in LL, LD12:12, and LD2:2. Densitometry of these blots is shown in Figure 1. (C) Persistence of 24 h circadian rhythm of luminescence in LD2:2 conditions. Upper panel, growth curve for cells in LD2:2. Lower panel, samples taken from the middle of the light interval or the middle of the dark interval in LD2:2 show robust luminescence rhythms. Before release into LD2:2 conditions, cells were given two LD12:12 cycles. To measure the luminescence of cultures from the batch flasks under LD2:2 conditions, 1 ml of cell culture was manually removed in either the middle of the light portion of LD2:2 or the middle of the dark portion of LD2:2 and transferred to a 20 ml vial with a tube containing n-decanal to measure the luciferase activity using a luminometer (Femtomaster FB12, Zylux Corporation, Knoxville, TN, USA). The maximum luminescence level at each time point was plotted for both the middle light (solid square) and the middle dark (solid triangle) collection times. (0.63 MB PDF) [file pbio.1000394.s001.pdf]

**Fig. S2**

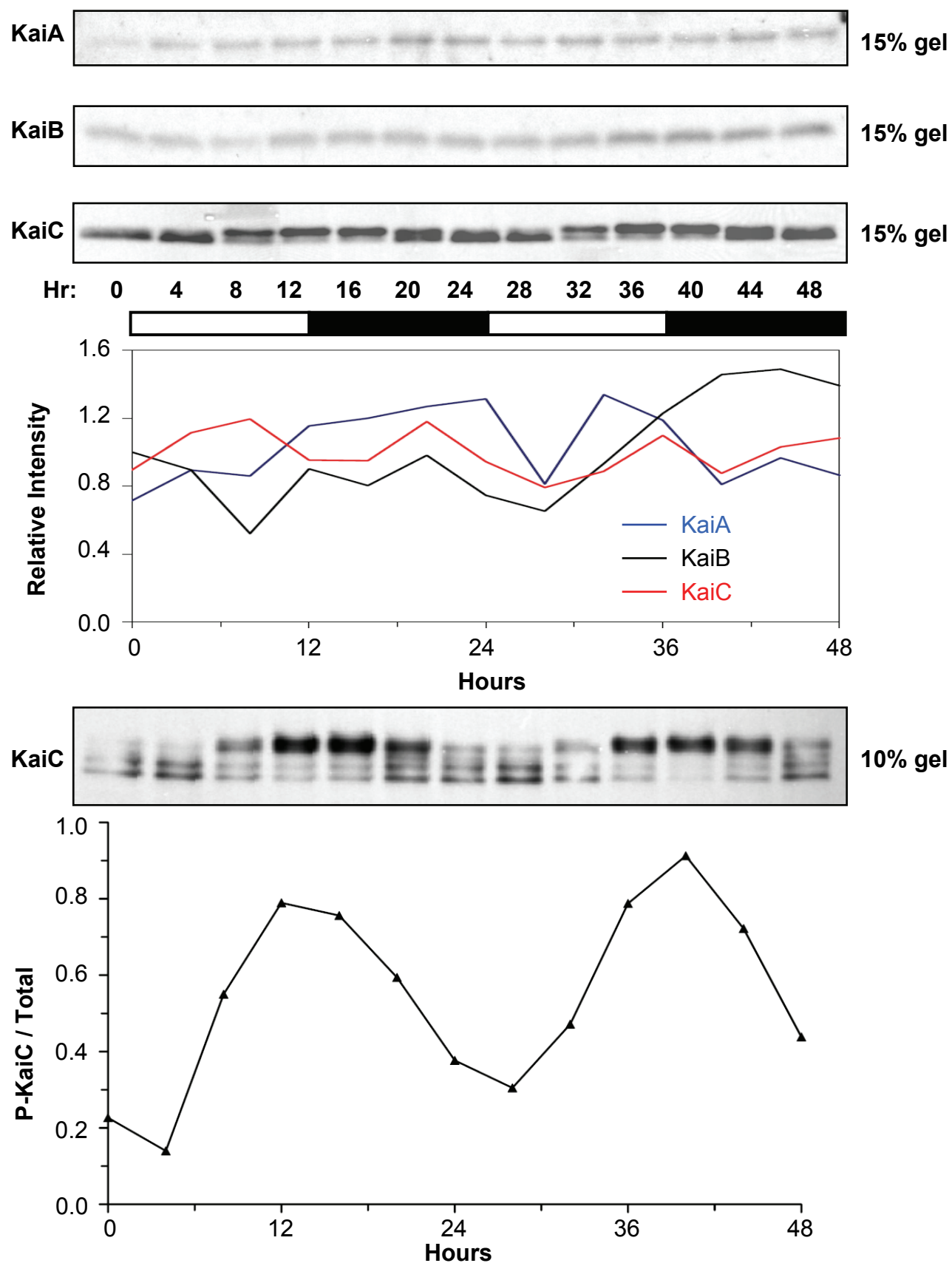

Supplement: Figure S2 — Absence of clear 24 h rhythmicity of abundances of KaiA, KaiB, and KaiC in LD12:12 despite the presence of a robust KaiC phosphorylation rhythm. A representative example is shown of an experiment where the phosphorylation rhythm was robust in LD12:12, while the abundances of the Kai proteins were not clearly rhythmic. Abundance data were collected from immunoblots run on 15% SDS-PAGE gels (to obtain a single protein band), whereas KaiC phosphorylation was determined on 10% SDS-PAGE gels (to separate the various KaiC phosphoforms)[4],[7],[20]. (0.18 MB PDF) [file pbio.1000394.s002.pdf]

Fig. S3

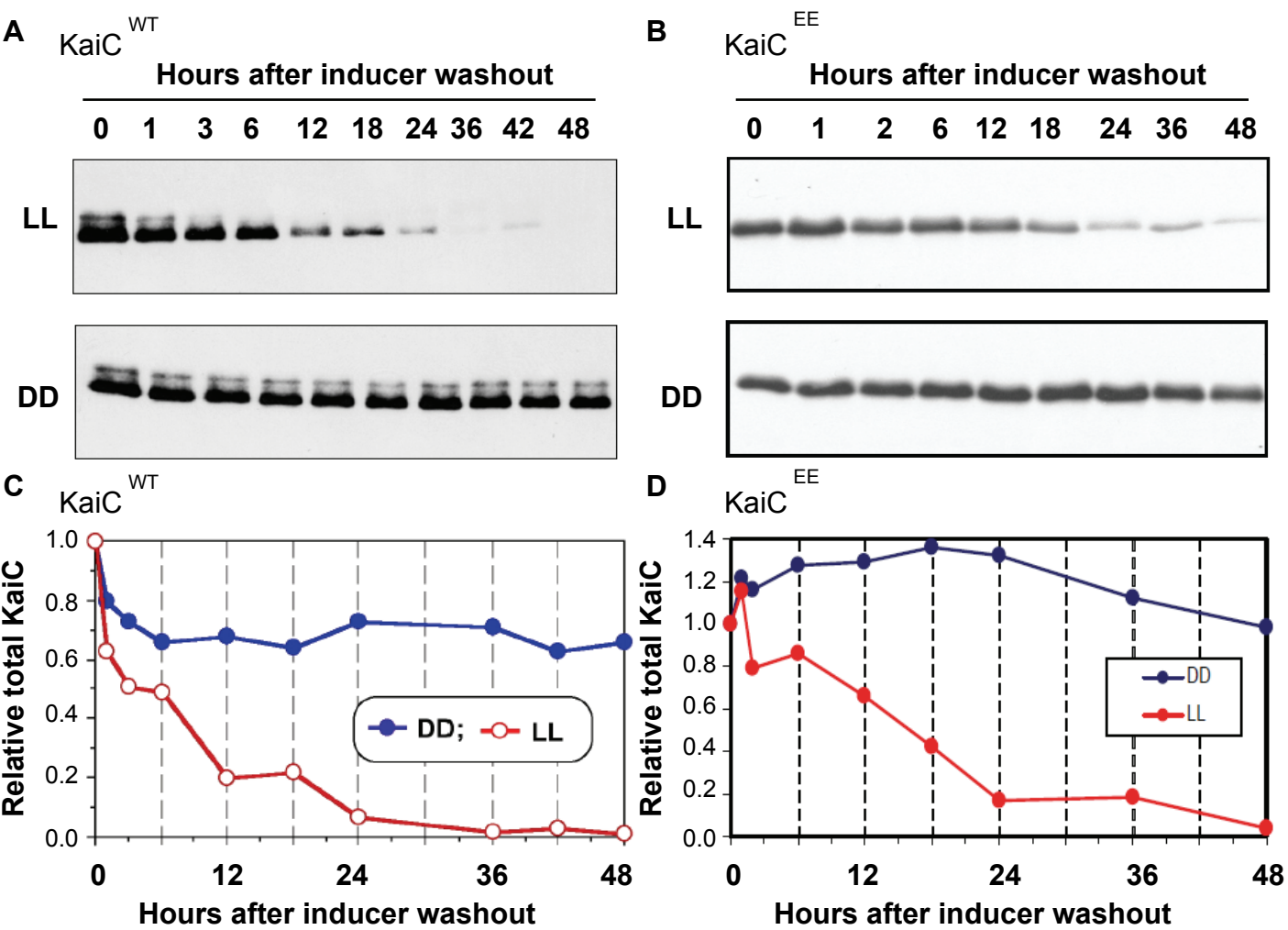

Supplement: Figure S3 — Degradation of KaiC protein is dependent upon light. (A) KaiCWT expression was induced in the KaiCOX strain by 100 µM IPTG for 6 h, and then the inducer was washed out and the cells were placed in either LL or DD. Samples were collected at the times indicated and processed for SDS-PAGE and immunoblotting as described previously for this type of degradation assay [7]. (B) Same as in panel A except with KaiCEE. (C, D) Quantification of the immunoblot data in panels A and B by Image J, which shows that the degradation of KaiCWT (panel C) and KaiCEE (panel D) in the cells proceeds in LL but is strongly inhibited in DD. (0.26 MB PDF) [file pbio.1000394.s003.pdf]

**Fig. S4**

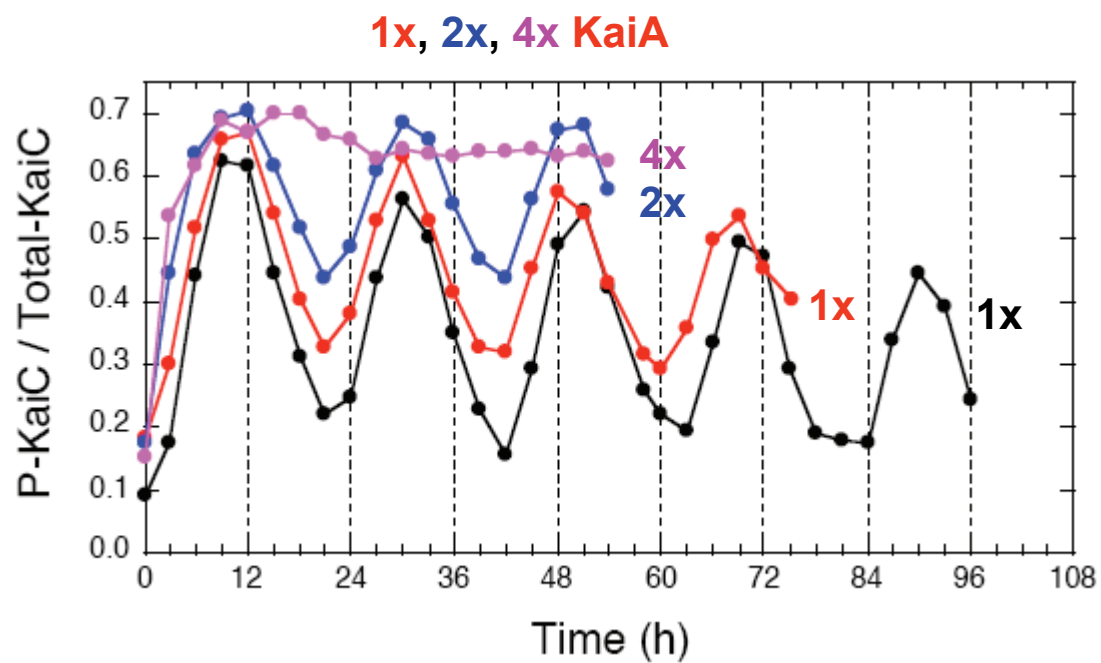

Supplement: Figure S4 — Elevated levels of KaiA in the in vitro reaction suppress the amplitude of the in vitro phosphorylation rhythm. KaiA, KaiB, and KaiC were mixed together at the final concentrations described in the Materials and Methods of Text S1 and were then dialyzed against medium without ATP for 24 h. The subsequent addition of 1 mM ATP initiates the in vitro rhythms. 1× KaiA = 50 ng/µl KaiA (duplicate reactions shown in red and black), 2× = 100 ng/µl KaiA (blue), and 4× = 200 ng/µl KaiA (purple). Elevated levels of KaiA cause hyper-phosphorylation of KaiA and suppression of the in vitro phosphorylation rhythm in the hyper-phosphorylated state. (0.05 MB PDF) [file pbio.1000394.s004.pdf]

**Fig. S5**

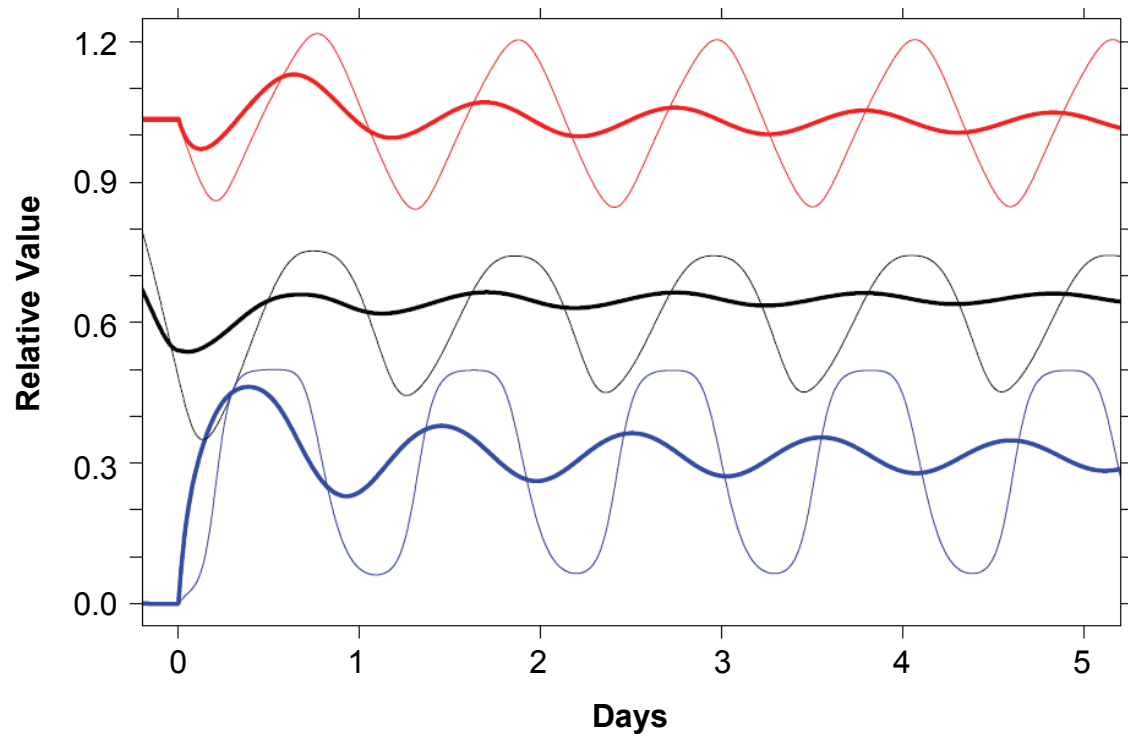

Supplement: Figure S5 — A simulated low amplitude KaiC phosphorylation rhythm (thick black trace) can be amplified into a larger amplitude rhythm in kaiBC mRNA (thick blue line) and Kai C protein (thick red line) abundance. The thin lines indicate the control simulation for the TTFL (Figure 5B in main text). (0.06 MB PDF) [file pbio.1000394.s005.pdf]

Fig. S6

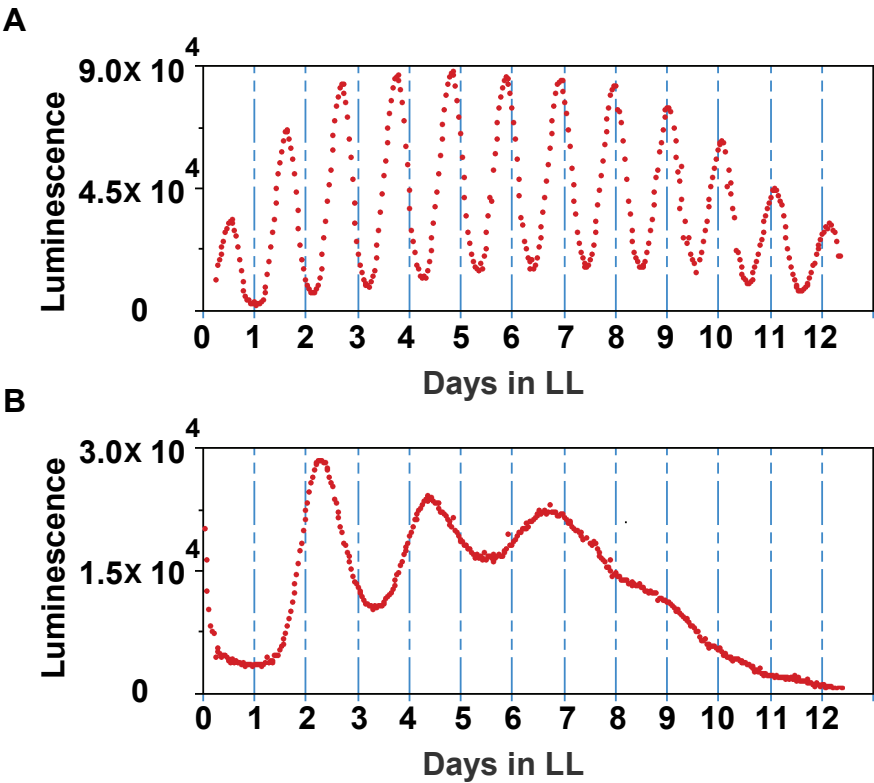

Supplement: Figure S6 — Another example of the rhythms expressed by KaiCWT (panel A) versus KaiCEE (panel B) strains at 30°C that illustrates the obvious damping of the KaiCEE strain. (0.05 MB PDF) [file pbio.1000394.s006.pdf]

**Fig. S7**

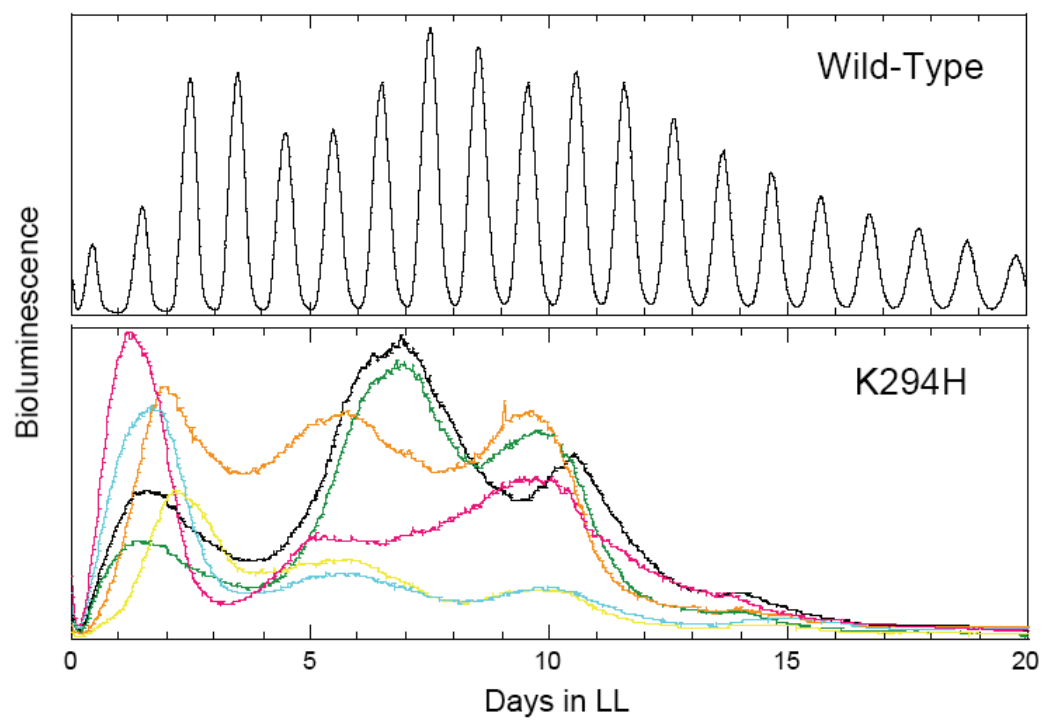

Supplement: Figure S7 — Strains expressing KaiCK294H are unstable with respect to phase, amplitude, and period. Upper panel is wild-type, and lower panel depicts a simultaneous recording of luminescence emitted by cells expressing KaiCK294H (KaiCK294H was constructed and expressed as in [16]). (0.04 MB PDF) [file pbio.1000394.s007.pdf]

**Fig. S8**

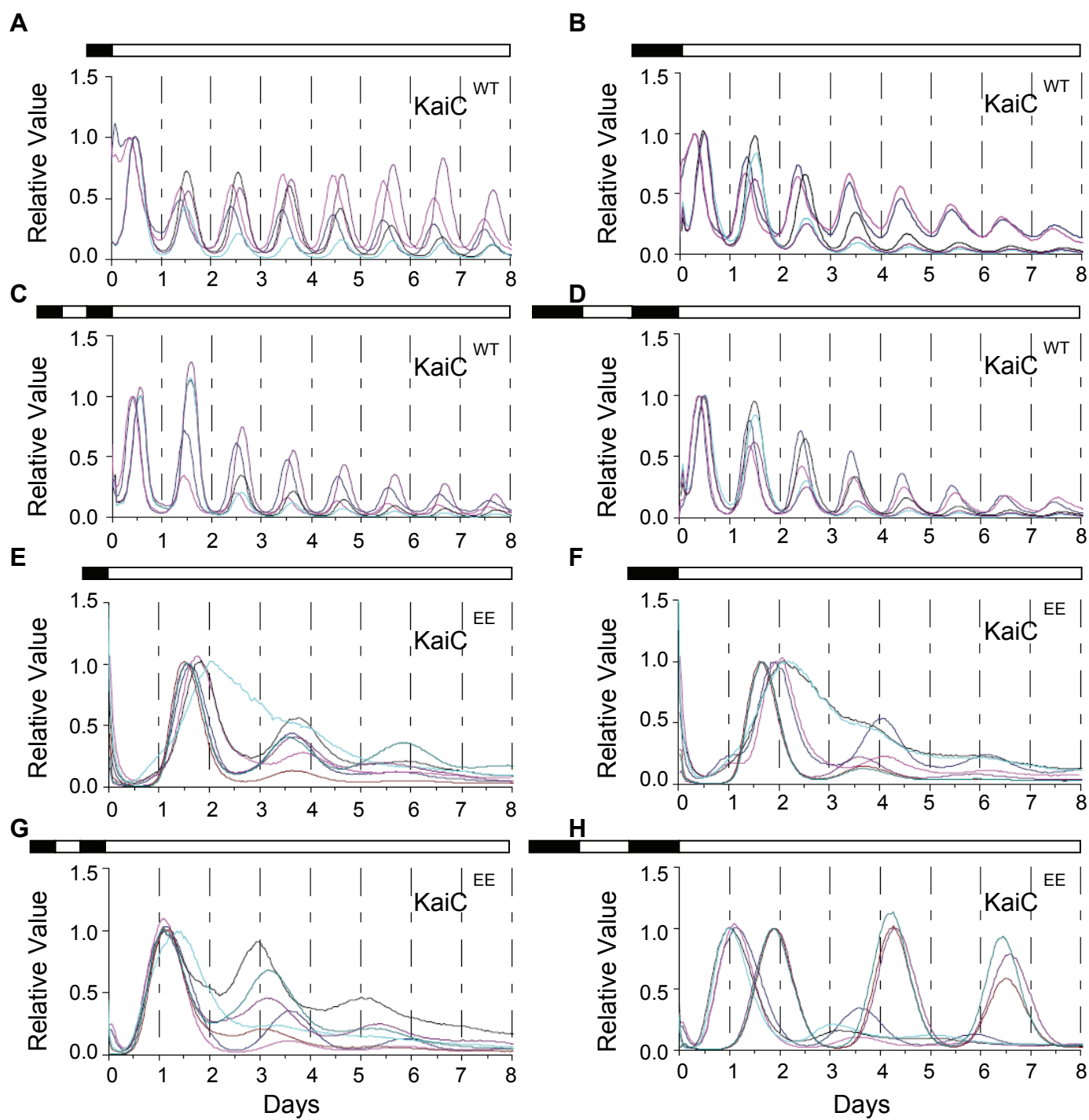

Supplement: Figure S8 — Raw data for Figure 4 (“Prior entrainment conditions determine the rate of damping in cells expressing KaiCEE”). Luminescence rhythms measured in vivo are shown for the WT strain (panels A–D) and for the KaiCEE strain (panels E–H). Cells were in LL at 30°C before and after the following entrainment conditions: (A, E) One 12 h dark pulse; (B, F) one 24 h dark pulse; (C, G) two 12 h dark pulses separated by one 12 h light pulse (i.e., 1.5 cycles of LD12:12); and (D, H) two 24 h dark pulses separated by one 24 h light pulse (i.e., 1.5 cycles of LD24:24). Each differently colored trace is from an independent measurement; n = 5 for each of the KaiCWT sample sets and n = 7 for each of the KaiCEE sample sets. (0.21 MB PDF) [file pbio.1000394.s008.pdf]

Fig. S9

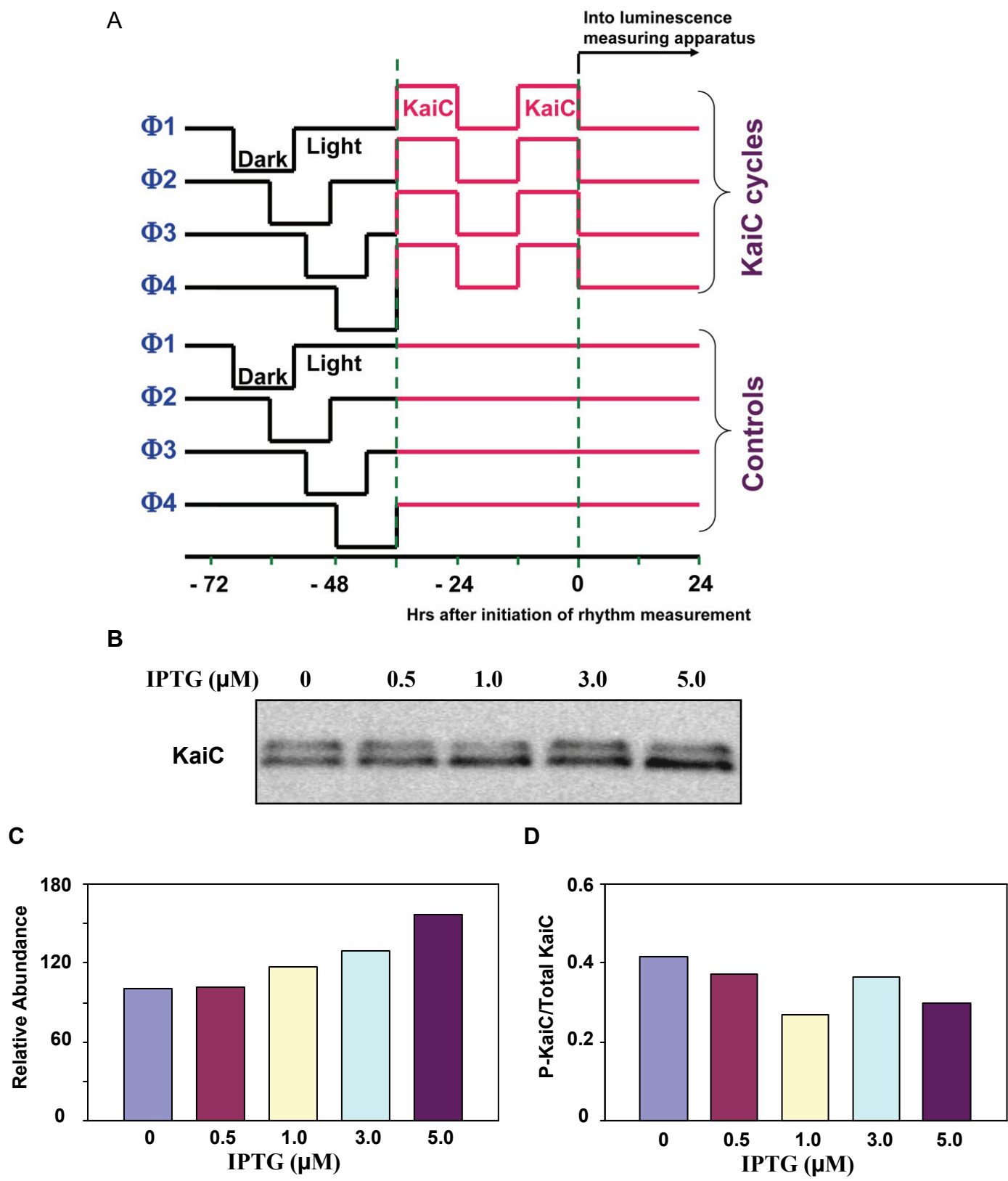

Supplement: Figure S9 — (A) Protocol of the phase locking experiment depicted in Figure 6B . Four separate cultures of the KaiCOX strain were phased to four different phases (Φ1, Φ2, Φ3, and Φ4) by exposure to 12 h dark pulses that were phased 6 h apart (at clock times 00:00, 06:00, 12:00, and 18:00). For the “Controls” cultures, no IPTG cycle was given prior to release into LL and initiation of the measurement of luminescence rhythms at time 0. The experimental “KaiC cycles” samples were exposed to two 12 h administrations of 5 µM IPTG, separated by a 12 h interval of medium without IPTG, thereby creating two 12:12 cycles of IPTG:no-IPTG. (B) Induction of new KaiC synthesis in strain KaiCOX by various concentrations of IPTG leads to changes in KaiC phosphorylation status and abundance. An immunoblot of KaiC is shown in panel B. (C) Densitometry of total KaiC abundance in the blot depicted in panel B. (D) Ratio of hyper-phosphorylated to total KaiC in the blot depicted in panel B. Data were analyzed by Image J. (0.31 MB PDF) [file pbio.1000394.s009.pdf]
