# Supplementary material for: Coupling of a Core Post-Translational Pacemaker to a Slave Transcription/Translation Feedback Loop in a Circadian System
Source: PLoS Biol. 2010 Jun 15;8(6):e1000394. doi: 10.1371/journal.pbio.1000394 (PMC2885980; doi:10.1371/journal.pbio.1000394)
Supplement: Text S1 — includes a complete description of the mathematical modeling, supplemental methods, and references for the Supporting Information. (0.11 MB DOC) [file pbio.1000394.s010.doc]

**Supporting Information (Text SI)**

**Supplemental Materials and Methods**

**Strains and culture conditions**

The wild-type reporter strain (AMC149) of the cyanobacterium *Synechococcus elongatus* PCC 7942 harbors luciferase reporter constructs, either *psbAIp::luxAB* or *kaiBCp::luxAB*. In either reporter, the expression of the *Vibrio harveyi* luciferase gene cassette *luxAB* is driven by the *psbA1* [Kondo *et al*., 1993] or the *kaiBC* promoter [Xu *et al*., 2003]. Luminescence rhythms from the *psbAIp::luxAB* or *kaiBCp::luxAB* reporters are approximately equivalent in both phase and intensity. Strain KaiA^OX^ has a *kaiBCp::luxAB* reporter (spectinomycin resistance marker in NS I) in which additional expression of wild-type *kaiA* is under the control of an IPTG-derepressible heterologous *trc* promoter, *trc*p*::kaiA* (kanamycin resistance marker in NS II)[Kutsuna *et al.,* 2007]. Strain KaiC^OX^ has a *psbAI*p*::luxAB* reporter (spectinomycin resistance marker in NS I) and *trc*p*::kaiC^WT^* (kanamycin resistance marker in NS II)[Xu *et al.,* 2000]. KaiC^EE^ is a *kaiBC*p::*luxAB* reporter expressing the double mutant KaiC^S431E/T432E^ [Xu *et al.,* 2009]. For measurement of KaiC degradation rate, strain ΔKaiC/*trc*p*::kaiC^WT^* was used, which is a *kaiC*-null strain with *trc*p*::kaiC^WT^* in NS II [Xu *et al*., 2003]. All strains were grown in modified BG-11 medium [Bustos & Golden, 1991] containing appropriate antibiotics with air bubbling of liquid cultures or on agar plates for solid cultures at 30ºC.

***In vivo* rhythm measurement and experimental overexpression of KaiA**

For assay of *in vivo* rhythms, cells were grown in constant light (LL; cool-white fluorescence at 40-50 μE/m^2^s). Before release to LL for assay, 1 ~ 2 light:dark cycles (e.g. LD 12:12) were given to synchronize the cells in the population. Luminescence rhythm measurements *in vivo* were performed as described previously [Xu *et al*., 2000]. For experimental overexpression of KaiA, KaiA^OX^ was grown in liquid BG-11 medium at 30ºC in LL until an OD_750_ of between 0.6-0.8 was reached. Then the cells were treated with two cycles of LD12:12, and IPTG was added 6 h before the beginning of the second 12 h dark interval.

Analysis of damping and bandwidth was performed with the LumiCycle data analysis program (Actimetrics, Evanston IL, courtesy of Dr. David Ferster). The program fits the data to a sine wave multiplied by an exponential decay factor. The damping rate (d) is the time constant of the following exponential fit:

L = A {[sin(2πf + Φ)][exp(-t/d)]}

Where d is the damping rate, L is the luminescence (counts/min), A is the amplitude, f is the frequency of the sine wave, Φ is the phase of the oscillation, and t is time. The data are fit to a low-order polynomial to get a baseline, which is then subtracted from the raw data. A Fourier Transform is performed to find the dominant frequency and phase. The timepoints for the peaks and troughs of the dominant sine wave are taken from the baseline-subtracted data, and the timepoints are then fitted to an exponential decay, which gives the amplitude (A) and the time constant of the damping (d). Damping rate (d) is the number of days required for the amplitude of the rhythm to decrease to 1/e (≈ 36.79 %) of the starting value.

**Determination of KaiC abundance and phosphorylation levels**

For the experiments of Figure 1, *S. elongatus* cells were harvested every 4 h for the LL and LD12:12 conditions and every 1 h for the LD2:2 conditions. Total protein was extracted as previously described [Xu *et al.,* 2000]. Protein concentration was determined by the Lowry method using BSA as a standard, and equal amounts of proteins were loaded into each well for SDS-PAGE and transfer to nitrocellulose membranes. The blots were treated with anti-KaiC serum, and detected by enhanced chemiluminescence (Pierce, Rockford, USA). KaiC protein abundance was determined on 15% SDS-PAGE gels (to obtain a single KaiC band), whereas KaiC phosphorylation was determined on 10% SDS-PAGE gels (to separate the various KaiC phosphoforms). Gel size was 16 cm X 16 cm X 1 mm, with electrophoresis at 4 ºC for 4-5 h at a constant current of 35 mA per gel. Gel images were analyzed by NIH Image J software.

**Phase locking by cycles of low levels of KaiC**

Cultures of KaiC^OX^ were inoculated onto nitrocellulose (NC) membranes placed on the surface of BG-11 agar plates containing appropriate antibiotics. After 5 d of growth in LL, these NC membrane cultures were divided into four groups with duplicates, and they were entrained with two LD12:12 cycles. There were a total of 4 different phasings of the LD12:12 cycles (Φ1, Φ2, Φ3, and Φ4) that were different from each other by 6 h (i.e., starting at laboratory clock time 06:00, 12:00, 18:00, and 00:00). After the final dark interval of the last group of plates (Φ4), the cultures were transferred back and forth between pre-warmed fresh BG-11 agar plates containing 0 or 5 μM IPTG for two cycles of: 12 h no IPTG followed by 12 h IPTG, thereby creating an experimentally controlled 12h:12h cycle of new KaiC synthesis (the parallel control cultures were transferred every 12 h between plates that had no IPTG). See Fig. S9A for an illustration of this protocol. We determined that a concentration of 5 μM IPTG increases KaiC abundance within cells by only ~40-50% above basal levels (Fig. S9C). After the final IPTG cycle, measurement of the luminescence rhythms in the cultures from each of these phases was performed in LL as previously described [Xu *et al.,* 2003].

**Preparation of Kai proteins and *in vitro* reactions**

Kai proteins from *S.* *elongatus* were expressed in *Escherichia coli* and purified as described previously [Mori *et al.,* 2007]. *In vitro* reactions were carried out at 30ºC in reaction buffer (20 mM Tris-HCl (pH 8.0), 150 mM NaCl, 5 mM MgCl_2_, 1mM ATP, 0.5 mM EDTA) using standard Kai protein concentrations: 50 ng/µl KaiA, 50 ng/µl KaiB and 200 ng/µl KaiC. The reaction mixture was dialyzed against this reaction buffer without ATP at 30 ºC for 24 h. The reaction was initiated by addition of 1mM ATP at 30 ºC. Extra KaiA protein (2X and 4X) was added as indicated (i.e., 2X = 100 ng/µl KaiA, and 4X = 200 ng/µl KaiA). Sample collections and analysis were as described previously [Mori *et al*., 2007].

**Mathematical Model Description**

**I. Post-Translational Oscillator/Constant Darkness (PTO/DD) Model**

KaiC hexamer concentrations are labeled by net population phosphorylation levels, Ci, where i = 0,N. To implement the model in its current form we used N =6 (for a total of 7 states). KaiA dimers are indicated by A(t) and KaiB tetramers by B(t). We neglect specific site-dependent modeling of KaiC phosphorylation (on residues S431 and T432 [Nishiwaki *et al*., 2007; Rust *et al.,* 2007]) and treat the system phenomenologically to study the effect of the transcription-translation feedback loop (TTFL) on the oscillations of phosphorylation in the KaiC population. The model reactions are as follows:

*Phosphorylation and de-phosphorylation:*

Auto-phosphorylation/dephosphorylation : Ci ↔ Ci+1

KaiA-KaiC phosphorylation//dephosphorylation: ACi ↔ ACi+1

KaiB-KaiC, KaiA-KaiB-KaiC

phosphorylation/dephosphorylation: BCi +1 ↔ BCi , ABCi+1 ↔ ABCi

*Hexamer Reactions:*

KaiA association (and dissociation): A + Ci ↔ ACi

A + BCi ↔ ABCi

KaiB association above threshold phosphorylation: B + Ci ↔ BCi (i > N)

Dissociation of KaiA-KaiB-KaiC complexes: BAC0 → B + A + C_0_

BC0 → B + C_0_

Monomer Exchange (i + j = k+m): Ci + Cj ↔ Ck + Cm

ACi + ACj ↔ ACk + ACm

ABCi + ABCj ↔ ABCk + ABCm

PTO Differential Equations: (i =1,N-1)

KaiC hexamers alone (k2 >> k_1_)

1.1 dC_0_/dt = -k_1_ C_0_ + k_2_ C1 - kA A*C0 + k-A (AC_0_) + kd (ABC_0_) + kd (BC_0_)

1.2 dCi/dt = k1 (Ci-1 - Ci) + k_2_ (Ci+1 - Ci ) - kA A*Ci + k-A (ACi)

1.3 dCN/dt = k_1_ CN-1 - k_2_ CN - kB B*CN + k-B (BCN) - kA A*CN + k-A (ACN)

KaiA-KaiC hexamers (k3 >> k_4_)

1.4 d(AC_0_)/dt = -k_3_ (AC_0_) + k_4_ (AC_1_) + kA A*C0 - k-A (AC_0_)

1.5 d(ACi)/dt = k3 ( (ACi-1) - (ACi) ) + k_4_ ( (ACi+1) - (ACi) ) + kA A*Ci - k-A (ACi)

1.6 d(ACN)/dt = k_3_ (ACN-1) - k_4_ (ACN )+ kA A*CN - k-A (ACN)

KaiB-KaiC (k_2_ >> k_1_)

1.7 d(BCN)/dt = kB B*CN - k-B (BCN) - k_2_ (BCN ) + k_1_ (BCN-1) - kA A*(BCN )

1.8 d(BCi)/dt = k1 ( (BCi-1) - (BCi) ) + k_2_ ((BCi+1) - (BCi) ) - kA A*(BCi )

1.9 d(BC_0_)/dt = -k_1_ (BC_0_) + k_2_ (BC_1_) - kd (BC_0_) - kA A*(BC_0_ )

KaiA-KaiB-KaiC (k_2_ >> k_1_)

1.10 d(ABCN)/dt = kA A*(BCN )- k_2_ (ABCN ) + k_1_ (ABCN-1)

1.11 d(ABCi)/dt = k1 ( (ABCi-1) - (ABCi) ) + k_2_ ((ABCi+1) - (ABCi) ) + kA A*(BCi )

1.12 d(ABC_0_)/dt = -k_1_ (ABC_0_) + k_2_ (ABC_1_) - kd (ABC_0_) + kA A*(BC_0_ )

KaiA (kA >> k-A)

1.13 dA/dt = - kA A*(Σ Ci ) - kA A*(Σ (BCi) )+ k-A Σ (ACi) + kd (ABC_0_)

KaiB (kB >> k-B)

1.14 dB/dt = -kB B*CN + k-B (BCN ) + kd ( (ABC0 )+ (BC_0_) )

*Monomer Exchange*

Monomer exchange is approximated phenomenologically by the reaction Ci +Cj → Ci+1 + Cj-1 (j >i) that acts to equalize the population concentration levels of phosphorylation by transfer of phosphates from hexamers with more phosphates (j) to hexamers with lower numbers of phosphates (i), j > i. Letting xk = Ck, (ACk), (BCk), or (ABCk):

1.15 dxi/dt = dxi/dt - ke xi*xj (j = i+1, N)

dxj/dt = dxj/dt - ke xi*xj

dxi+1/dt = dxi+1/dt + ke xi*xj

dxj-1/dt = dxj-1/dt + ke xi*xj

*PTO Initial Conditions and rates*

All the differential equations are scaled to the initial KaiC concentration, C_0_ (t=0) so that the fraction of each is followed with respect to time.

***Simplified KaiA Sequesteration Model with Monomer exchange*.** We introduce a simplified model that neglects the auto-phosphorylation/dephosphorylation reactions of KaiC (1.1 -1.3) and corresponding KaiB•KaiC complex formation reactions (1.7-1.9) and instead considers the approximate complex formation and phosphorylation reaction sequence:

1.16 C_0_ → AC0 → AC_1_...→ACN → ABCN → ABCN -1→... → ABC_0_ → A+B+C_0_.

Monomer exchange occurs among A•C complexes (phosphorylation phase) and among A•B•C complexes (dephosphorylation phase) but not between A•C complexes and A•B•C complexes. The dynamics of this model are very similar to the full model in most simulations and provide a simpler interpretation of the resulting dynamics. However, the full model is required to adequately represent the time dependence of complex abundances reported previously [Kageyama *et al.,* 2006; Mori *et al.,* 2007]. For the text figures we have used the simplified model.

**II. In Vivo Model (LL)**

The *in vivo* model consists of the PTO and a generic Transcription-Translation Feedback Loop (TTFL) in which KaiC negatively represses its own production as indicated experimentally by overexpression of KaiC and its effect on *kaiBC* promoter activity. The precise form of this repression in terms of the various states of KaiC (C_i_, BC_i_, AC_i_, ABC_i_) however, is not derivable from existing experimental data. The mRNA abundance is modeled as follows (a TTFL based on Goldbeter 1995 PER model [Goldbeter, 1995]) and includes a nonlinear repression term and a linear degradation rate:

1.17 dC_mRNA_/dt = k_+,mRNA_ 1/(1 +(f(x)/K)^n^ ) - k-,_mRNA_ C_mRNA_

where n is the coefficient of cooperativity for repression, K is a threshold constant for repression, and x represents hexamer states C_i_, (AC_i_), (BC_i_), or (ABC_i_). The function f(x) is assumed to be some linear combination of these states: f(x) = S {a_i_ C_i_ + b_i_ (BC_i_) + c_i_ (ABC_i_) + d_i_ (AC_i_)} parameterized by constant coefficients. An assumption consistent with current data is that KaiB•KaiC and KaiA•KaiB•KaiC (BC_i_ and ABC_i_) complexes suppress promoter activity. The relative phase of mRNA, protein, and phosphorylation in LL is determined by the form(s) of KaiC that suppress(es) transcription. Simulations indicate that phase relationships of mRNA, protein, and phosphorylation consistent with previously published studies are obtained when hyper-phosphorylated KaiC states (including the KaiB•KaiC and KaiA•KaiB•KaiC complexes) are primarily responsible for mRNA suppression.

In LL the proteins KaiC_0_ and KaiB are translated from mRNA and are also degraded so that Equations 1.1 and 1.14 are modified to include

1.18 dC_0_/dt = ...+ k_trans_ C_mRNA_ - k_deg_ C_0_

1.19 dB/dt = ...+ k_trans_ C_mRNA_ - k_deg_ B

The other complexes are likewise assumed to be degraded (which might be complex-dependent and phosphorylation-dependent). For simplicity we will assume constant and equal degradation rates for all forms of KaiC (y = AC_i_, ABC_i_):

1.20 dy/dt = ... -k_deg_ y

Since KaiA abundance shows little variation in abundance in LL we introduce an *ad hoc* production term for KaiA in the simplified model (modeled as if KaiAC_i_ → KaiA + KaiC_i_ and the KaiC_i_ is degraded; this is not necessary in the full DD model as C_i_ states are included).

1.21 dA/dt = ...+ k_deg_ y

**III. KaiC^EE^ Model**

The mutant protein KaiC^EE^ is a phosphomimetic for a constitutively hyperphosphorylated KaiC. Since there are no intermediate phosphorylation states nor a functional PTO the model simplifies considerably:

dC_EE_/dt = k_-B_ (BC^EE^) – k_B_ B*C^EE^ + k_trans_ C^EE^_mRNA_ - k_deg_ C^EE^

dB/dt = -k_B_ B*C^EE^ + k-_B_ (BC^EE^) + k_trans_ C^EE^_mRNA_ - k_deg_ B

dC^EE^_mRNA_/dt = k_+,mRNA_ 1/(1 +(BC^EE^/K)^n^ ) - k-,_mRNA_ C^EE^_mRNA_

d(BC^EE^)/dt = k_B_ B*C^EE^ - k-_B_ (BC^EE^) - k_deg_ (BC^EE^)

Here the repression function f(x) is simply B•C^EE^ consistent with the simulation results for the wild type system simulations. We rescale as before to KaiC^EE^(t=0) with all other variables relative to this scale and B(0) = C^EE^(0). This model does not show sustained oscillations (n = 4) but can produce long period decaying oscillations to a steady-state.

**IV. Figure Rates and Parameters**

Figures 5 (A, C and E) use the PTO parameters given below.

Figures 3C, 5 (B,D and F) and Fig 6 use the PTO and TTFL parameters given below.

*PTO/DD Simplified Model*

phosphorylation (hr ^-1^) dephosphorylation(hr ^-1^)

+ KaiA k_3_ = 0.6 k_4_ =0.0

+ KaiB k_1_ = 0.0 k_2_ = 0.6

KaiA Association k_A_ = 5.0 (μM^-1^ hr ^-1^)

KaiA Dissociation k _-A_ = 0.0 (hr ^-1^)

KaiB Threshold N = 6

KaiB Association k _B_ = 0.5 (μM^-1^ hr ^-1^)

KaiB Dissociation k _-B_ =0.0 (hr ^-1^)

KaiA-KaiB-KaiC Dissociation: k_d_ = 1.0 (hr ^-1^)

phosphorylation dephosphorylation

Monomer Exchange (μM^-1^ hr ^-1^) k_e1_ =2.5 k_e2_ = 5.0

*TTFL* (rates are hr ^-1^)

Concentrations scaled to initial KaiC_0_ (t=0) concentration. C_mRNA_(t=0) = 1.0

mRNA synthesis rate: k+,mRNA = 0.5

mRNA degradation rate: k-,mRNA = 1.0

KaiC_0_ and KaiB synthesis rate: ktrans= 0.5

KaiC/KaiA•KaiC/ KaiA•KaiB•KaiC degradation rate: kdeg = 0.05

Suppression: dC_mRNA_/dt = k_+,mRNA_ 1/(1 +(f(x)/K)^n^ ) - k-,_mRNA_ C_mRNA_

K = 1

n = 4

f(x) = Σ(ABC)_i_ (KaiA•KaiB•KaiC complexes suppress transcription)

Noise simulations (Fig 5C and D)

Noise was explicitly introduced by modifying the KaiC_0_ concentration using Eqn (1.1) to include a term with production or degradation specified by a uniform random number on [-1,1] and suitably re-scaled.

Phase simulations (Fig 5 D and E, Fig 6A)

KaiC_0_ concentration was varied by adding a pure oscillatory term to Eqn (1.1), 0.2 ω cos(ωt + ϕ) with an angular frequency of ω = (2π/24) hr^-1^, where ϕ is the phase.

*KaiC-EE Simulation Rates: Fig 3D*

TTFL (rates are hr ^-1^)

TTFL Suppression: dC_mRNA_/dt = k_+,mRNA_ 1/(1 +(f(x)/K)^n^ ) - k-,_mRNA_ C_mRNA_

K = 1

n = 4

f(x) = BC_EE_ (KaiB•KaiC-EE complexes suppress transcription)

BC_EE_ association (μM^-1^ hr ^-1^): 0.02

BC_EE_ dissociation (hr ^-1^): 0.01

*Parameter Set #1 (black trace)*

C_mRNA_(t=0) = 1.0

B/C_EE_ (t=0)= 1.5

mRNA synthesis rate: k+,mRNA = 0.5

mRNA degradation rate: k-,mRNA = 0.01

KaiC_0_ and KaiB synthesis rate: ktrans= 0.2

KaiC/KaiA•KaiC/ KaiA•KaiB•KaiC degradation rate: kdeg = 0.05

*Parameter Set #2 (red trace)*

C_mRNA_(t=0) = 1.0

B/C_EE_ (t=0)= 1.0

mRNA synthesis rate: k+,mRNA = 0.1

mRNA degradation rate: k-,mRNA = 0.05

KaiC_0_ and KaiB synthesis rate: ktrans= 0.2

KaiC/KaiA•KaiC/ KaiA•KaiB•KaiC degradation rate: kdeg = 0.05

*Supplemental Figure S5:*

The parameters are the TTFL control parameters from Fig 5B except the KaiB degradation rate was increased by a factor of 3.8 in the simplified model to mimic a hyperphosphorylated KaiC state.

**References for Supporting Information**

Bustos SA, Golden SS (1991) Expression of the psbDII gene in Synechococcus sp. strain PCC 7942 requires sequences downstream of the transcription start site. J Bacteriol 173: 7525-7533.

Goldbeter A (1995) A Model for Circadian Oscillations in the Drosophila Period Protein (PER). Proc Biol Sci 261: 319-324.

Kageyama H, Nishiwaki T, Nakajima M, Iwasaki H, Oyama T, et al. (2006) Cyanobacterial circadian pacemaker: Kai protein complex dynamics in the KaiC phosphorylation cycle in vitro. Mol Cell 23: 161-171.

Kitayama Y, Nishiwaki T, Terauchi K, Kondo T (2008) Dual KaiC-based oscillations constitute the circadian system of cyanobacteria. Genes Dev 22: 1513-1521.

Kondo T, Strayer CA, Kulkarni RD, Taylor W, Ishiura M, et al. (1993) Circadian rhythms in prokaryotes: luciferase as a reporter of circadian gene expression in cyanobacteria. Proc Natl Acad Sci U S A 90: 5672-5676.

Kutsuna S, Kondo T, Ikegami H, Uzumaki T, Katayama M, et al. (2007) The circadian clock-related gene pex regulates a negative cis element in the kaiA promoter region. J Bacteriol 189: 7690-7696.

Mori T, Williams DR, Byrne MO, Qin X, Egli M, et al. (2007) Elucidating the ticking of an in vitro circadian clockwork. PLoS Biol 5: e93.

Nishiwaki T, Satomi Y, Kitayama Y, Terauchi K, Kiyohara R, et al. (2007) A sequential program of dual phosphorylation of KaiC as a basis for circadian rhythm in cyanobacteria. EMBO J 26: 4029-4037.

Rust MJ, Markson JS, Lane WS, Fisher DS, O'Shea EK (2007) Ordered phosphorylation governs oscillation of a three-protein circadian clock. Science 318: 809-812.

Xu Y, Mori T, Johnson CH (2000) Circadian clock-protein expression in cyanobacteria: rhythms and phase setting. EMBO J 19: 3349-3357.

Xu Y, Mori T, Johnson CH (2003) Cyanobacterial circadian clockwork: roles of KaiA, KaiB and the kaiBC promoter in regulating KaiC. EMBO J 22: 2117-2126.

Xu Y, Mori T, Qin X, Yan H, Egli M, Johnson CH (2009) Intramolecular Regulation of Phosphorylation Status of the Circadian Clock Protein KaiC. PLoS ONE 4: e7509.
